# Prevalence and factors associated with depression and anxiety in patients with chronic kidney disease

**DOI:** 10.12688/f1000research.178880.2

**Published:** 2026-07-03

**Authors:** Evelyn Goicochea Rios, Ana Chian Garcia, Jhony Paredes Silva, Jessica Vicuña Villacorta

**Affiliations:** 1 La Libertad, Universidad Cesar Vallejo Facultad de Ciencias Medicas, Trujillo, La Libertad, 13001, Peru

**Keywords:** Anxiety, depression, hemodyalisis, peritoneal dyalisis, uremic toxins, HEADS, diabetes mellitus, hypertension

## Abstract

**Background:**

Depression and anxiety are common in chronic kidney disease (CKD) and can increase morbidity and mortality. Therefore, the objective was to analyze their prevalence and the associated demographic, clinical, and laboratory factors in CKD patients undergoing dialysis.

**Methodology:**

Correlational design research with prospective cohort. Sample of 107 patients, 67 on hemodialysis and 40 on peritoneal dialysis (PD). The Hospital Anxiety and Depression Scale questionnaire was used after obtaining free and informed consent. The Spearman correlation coefficient, Chi ^2^ and multivariate ordinal logistic regression analysis were used.

**Results:**

Females predominated. Hemodialysis patients were mostly single (47.8%), unemployed (44.8%), and had a basic education, while 60% of PD patients were married, 50% were employed, and 57.5% had a higher education. Hypertension and diabetes mellitus were the primary causes of CKD. In the PD group, 37.5% presented moderate-severe symptoms of anxiety, and in hemodialysis, 29.9%. Moderate-severe symptoms of depression were present in 41.8% of the hemodialysis group. One-third of men and women presented moderate-severe symptoms of anxiety, and 66.7% of illiterate individuals presented mild symptoms. A significant association was found between depression and employment status (sig < 0.05); urea (rho = −0.204; p = 0.036), hemoglobin (rho = −0.199; p = 0.041), and phosphorus (rho = −0.228; p = 0.019); and a significant inverse correlation between anxiety and hemoglobin (rho = −0.197; p = 0.042). The multivariate ordinal regression model showed an association between anxiety and older age, lower calcium levels, and single marital status.

**Conclusion:**

Early identification of symptoms of anxiety and depression in patients with CKD on dialysis is required to properly manage diagnosed cases.

## I. Introduction

Depression and anxiety disorders are prevalent in people with chronic kidney disease (CKD), especially in advanced stages and on dialysis. ^1^ It is estimated that depression affects 25% and anxiety 23% of dialysis patients and according to other studies, the prevalence can range from 27 to 30% ^2^ and from 69% to 71% respectively. ^3^

Depression is defined as feelings of sadness, guilt, disinterest, low self-esteem, insomnia, hyporexia/anorexia, fatigue, and lack of concentration and pleasure. ^4^ It has personal repercussions at the psychological, emotional, and behavioral levels, ^2, 5^ and affects treatment compliance, so detecting the factors associated with depression should be a priority. ^3, 5^ Risk factors for depression include being female, low socioeconomic status, stressful life events, lack of social support, serious or chronic illness, and a history of eating disorders. ^5^

Anxiety is characterized by feelings of uncertainty and fear and can manifest as palpitations, tremors, indigestion, nervousness, shortness of breath, and diaphoresis, often being confused with other pathologies. ^6^ However, anxiety disorders have been less studied in patients with advanced stages of CKD, and their impact is largely unknown in this population. ^1, 2^

Chronic kidney disease (CKD) is strongly associated with an increase in mental health problems, including depression and anxiety. ^1– 3^ There is also evidence of its association with central nervous system diseases, cognitive dysfunction, and dementia. People with CKD are more likely to have strokes and subclinical cerebrovascular disease than the general population. It has also been reported that uremic toxins related to kidney damage may predispose individuals to neurological disorders. ^7^

A systematic review covering information on 80,932 individuals with CKD from 27 countries found an overall prevalence of depression of 26.5% and 39.6% when using the International Classification of Diseases. Depression was significantly more common among individuals on chronic hemodialysis compared to pre-dialysis patients (29.9% and 18.5%, respectively). ^8^ Another study of 376 patients with CKD, a third of whom were on hemodialysis, found that 74% suffered from depression, with mild depression predominating (16%). Among the factors significantly associated with depression, they found the duration of CKD since diagnosis, the level of independence in performing routine activities at home, the ability to generate income, and lifestyle changes. ^9^

The association between depression and various sociodemographic variables in patients with CKD has been documented, ^10– 13^ including gender, educational attainment, age group, employment status and duration of CKD ^10^ and monthly income ^13^; other authors consider that family support, ^10, 11^ social support and marital status ^12^ are associated with clinical depression, as are a negative perception of the disease and low self-esteem. ^10^ It is also noted that socio-economic variables affect the quality of life of people with CKD. ^14^

Other studies with CKD patients report that single marital status and female gender ^13, 15^ were significantly associated with depression and anxiety, as well as unemployment and polypharmacy. ^15^ Bivariate and multivariate logistic regression in another study showed that social support and time since CKD diagnosis were significantly associated with depression and anxiety. Other associated factors include uric acid levels ^16^ and family history of mental illness, with patients with CKD and comorbidities being 1.7 times more likely to develop such mental disorders. ^13^

In Peru, most patients with CKD who require dialysis are treated by Essalud and the Integral Health Insurance system. From 2019 to 2023, 205,501 cases of CKD were diagnosed (2.16% of the total insured population), of which 10% were in stage G5 as confirmed by laboratory tests. ^17^ Current treatment modalities do not offer a cure, but they can alleviate symptoms and prolong the life of patients who come to dialysis with great fear because they associate this condition with mortality, radical changes in their lifestyle and personal routine, and uncertainty about the progression of this chronic disease. ^18^ No research on mental illnesses such as depression, anxiety, stress, and sleep disorders in CKD patients on dialysis was found in the Peruvian medical literature. Therefore, the objective of this study is to analyze the prevalence of depression and anxiety in CKD patients and the demographic, clinical, and laboratory factors associated with these pathologies. The findings will provide primary data on this issue in a systematic manner to adapt the protocols for the diagnosis and treatment of depression and anxiety in patients with CKD.

## II. Patients and methods

### Study design, population, and sample

A cross-sectional study was conducted. ^19^ The target population consisted of 160 adults patients diagnosed with CKD on dialysis treated between June and December 2025 at an Essalud Social Security hospital and at the SIS (Seguro Integral de Salud) in the province and district of Trujillo, Peru, who met the following inclusion criteria: signing the free and informed consent form, both sexes, with laboratory results, demographic data, clinical data, and complete records in the medical history. Two patients with incomplete medical records and one minor patient were excluded.

A stratified proportional probability sampling method was used, and the sample size was determined using the formula for finite populations ^19^ with a 95% confidence level and an adjusted error of 5.5%. It was then distributed proportionally in each stratum according to its relative size, comprising 40 patients on peritoneal dialysis and 67 patients on hemodialysis with a diagnosis of stage G5 CKD who met the above inclusion criteria.

### Data collection techniques and instruments

Interviews and document analysis were used. ^19^ Demographic data were recorded on a structured form designed by the researchers: age, sex, marital status, educational level, employment status; clinical data: years on dialysis, primary cause of CKD, weight, height, body mass index (BMI), frequency and type of dialysis, type and number of comorbidities, sleep disturbances, number of medications, and laboratory parameters: hemoglobin, serum albumin, creatinine, urea, blood glucose, ferritin, C-reactive protein, calcium, phosphorus, and parathyroid hormone. This form included the HADS, ^20^ a tool that has been validated in numerous studies as an effective instrument for identifying symptoms of anxiety and depression in diverse populations, including the general population, people with chronic or psychiatric conditions, ^21, 22^ and patients undergoing haemodialysis ^23^ and peritoneal dyalisis. ^20^ Designed to minimise the impact of somatic symptoms, such as headaches, chest discomfort, fatigue or sleep disturbances, it focuses on the emotional and cognitive aspects of anxiety and depression. ^24^ Is characterised by its good internal reliability and test-retest. ^20^ The psychometric properties of the HADS were assessed in a Peruvian study of patients with chronic kidney disease, revealing good internal consistency (overall McDonald’s omega ω = 0.806). The anxiety subscale showed acceptable reliability (ω = 0.757), whilst the depression subscale showed lower consistency (ω = 0.680). ^25^

HADS consists of 14 items related to the emotional and cognitive aspects of depression and anxiety: seven for each subscale. Each item is scored from 0 to 3, and the score ranges from 0 to 21 for each subscale, with a total score ranging from 0 to 42. Scores of 0–7 indicate no significant symptoms, 8–10 indicate mild symptoms, and 11–15 and 16–21 indicate moderate and severe symptoms, respectively. ^21^

The measure was implemented to all patients in the peritoneal dialysis and hemodialysis program. In the case of hemodialysis, patients were interviewed three times a week prior to treatment, depending on availability. In the case of peritoneal dialysis patients receiving home treatment, they were interviewed on the day they attended their outpatient nephrology appointment. The instrument was validated by nephrologists and family doctors. An Aiken V score was obtained, with a value of 0.94. ^26^

### Procedure

The research was authorized by the La Libertad Healthcare Network after review and approval by the Institutional Ethics Committee. The inclusion criteria were then applied to the target population that agreed to participate. Each patient was given an information sheet about the study and, after signing the free and informed consent form, the information from their medical records was collected in an anonymous database. The HADS scale was implemented during the interview. Subsequently, the information was statistically analyzed, and this article was prepared. All variables recorded by the responsible physician were considered, and the results were presented considering the STROBE checklist. ^27^

### Laboratory measurements

The blood samples were taken in accordance with the protocol established for sampling hemodialysis patients (before the start of the treatment session) and, in the case of peritoneal dialysis patients, when they attended the laboratory. The normal ranges for the tests are: serum albumin 3.8–5.0 g/dL; creatinine: women, 0.5–1.1 mg/dL and men, 0.6–1.2 mg/dL; serum urea 12–54 mg/dL ^28^; hemoglobin >12 g/dL for women and > 13 g/dL for men; ferritin 30–400 ng/mL for men and 13–150 ng/dL for women ^29^; serum calcium 8.5–10.5 mg/dL; serum phosphorus 2.5–4.5 mg/dL; parathyroid hormone 10–55 pg/mL; C-reactive protein <1.00 mg/dL and blood glucose 75–110 mg/dL. ^28^ In adults <60 years of age, overweight was defined as a BMI of 25–29.9 and obesity as a BMI >30; while in adults >60 years of age, overweight was considered if BMI 28–31.9 and obesity >32. ^30^

### Data processing

JAMOVI, ^31^ open-access statistical software, was used to record each patient’s data, excluding personal identification details. A database was generated in Excel. Continuous data are presented using means and standard deviation, and categorical data are presented as percentages. ^19^

### Statistical analysis

Descriptive statistics were used to analyze tables, averages, and standard deviations to analyze the behavior of the study variables. For the inferential analysis, the Spearman’s correlation coefficient and Chi ^2^ were used in the case of categorical variables, and multivariate ordinal logistic regression analysis ^32^ was performed to estimate the factors associated with depression and anxiety. A p-value <0.05 was considered statistically significant. ^32^

### Ethical considerations

The Declaration of Helsinki was applied about respect for the confidentiality and accuracy of the data collected during the study, which are presented faithfully. Authorship contributions and transparency in conflicts of interest were reported. ^32, 33^ The project was reviewed and approved by the Ethics Committees of both La Libertad Healthcare Network and the School of Medicine – UCV. (Certificate No. 29 issued by the Research Ethics Committee of La Libertad Healthcare Network on February 10, 2025, and Favorable Opinion of the Research Ethics Committee of Medicine – UCV on June 5, 2025. Approval was obtained from the ethics committees prior to initiating this study.

## III. Results

The Table 1 shows that females predominate in both groups (60% in peritoneal dialysis versus 52.2% in hemodialysis). Patients on peritoneal dialysis were married (60%), employed (50%), and had a higher level of education (57.5%). Patients on hemodialysis were single (47.8%), unemployed (44.8%), and had a primary or secondary level of education (37.3% for each level).

**Table 1.** Demographic and clinical profiles according to type of dialysis in patients with chronic kidney disease.

		Group			
		Haemodyalisis		Peritoneal dialysis	
		N°	%	N°	%
Sex	Male	32	47.8%	16	40.0%
	Female	35	52.2%	24	60.0%
Marital status	Single	32	47.8%	11	27.5%
	Married	23	34.3%	24	60.0%
	Divorced-Widowed	12	17.9%	5	12.5%
Employment status	Working	17	25.4%	20	50.0%
	Retired	18	26.9%	14	35.0%
	Unemployed	30	44.8%	2	5.0%
	Housewife	2	3.0%	4	10.0%
Educational level	Illiterate	3	4.5%	0	0.0%
	Primary	25	37.3%	4	10.0%
	Secondary	25	37.3%	8	20.0%
	Technical	3	4.5%	5	12.5%
	Higher	11	16.4%	23	57.5%
Primary cause of CKD	Diabetes Mellitus	21	31.3%	11	27.5%
	Hypertension	31	46.3%	17	42.5%
	Nephritis	2	3.0%	0	0.0%
	Glomerulonephritis	3	4.5%	2	5.0%
	Unknown	10	14.9%	5	12.5%
	Other	0	0.0%	5	12.5%
Comorbidities	Diabetes mellitus	4	6.0%	2	5.0%
	Heart disease/hyperten-sion	13	19.4%	19	47.5%
	Cancer	0	0.0%	0	0.0%
	Peripheral vascular disease	8	11.9%	3	7.5%
	Cerebral vascular disease	1	1.5%	1	2.5%
	Hepatitis C	0	0.0%	0	0.0%
	None	38	56.7%	8	20.0%
	Other	3	4.5%	7	17.5%
Sleep disorders	Yes	31	46.3%	28	70.0%
	No	36	53.7%	12	30.0%
N° of medicines	1–3	34	50.7%	9	22.5%
	4–6	31	46.3%	24	60.0%
	> 6	2	3.0%	7	17.5%
Anxiety according to HEADS score		7.885 ± 4.68		8.725 ± 4.72	
Depression according to HEADS score		9.833 ± 3.43		8.325 ± 4.21	

The main primary causes of CKD in both groups were hypertension and type 2 diabetes mellitus. In terms of comorbidities, more than half of the patients on hemodialysis had no associated diseases (56.7%), while in peritoneal dialysis, heart disease and hypertension were the most prevalent (47.5%).

Sleep disorders were more common in patients undergoing peritoneal dialysis (70%) than in those undergoing hemodialysis (46.3%), as was a higher drug burden (4 to 6 drugs), whereas in hemodialysis the use of 1 to 3 drugs was more common. Moderate to severe anxiety symptoms were more common in the peritoneal dialysis group (37.5%) than in the hemodialysis group (29.9%), while hemodialysis patients showed a higher proportion of moderate to severe depressive symptoms (41.8%).

In Table 2, 27.1% of men and 37.3% of women presented moderate to severe symptoms of anxiety. Most single people (41.9%) showed moderate to severe symptoms of anxiety, as well as 27.7% of married people and 23.5% of divorced or widowed people. According to educational level, one-third of patients at each level – except for those with technical qualifications – presented with moderate to severe anxiety symptoms. 50% of people whose occupation was ‘housewife’ also experienced moderate to severe anxiety symptoms; however no significant association was found between anxiety and sociodemographic characteristics (sig > 0.05).

**Table 2.** Depression and anxiety profiles and their association with demographic variables: gender, age, marital status, employment status, and educational level.

		Anxiety						
		No symptoms		Minor symptoms		Moderate - severe		
		N°	%	N°	%	N°	%	
Sex	Male	23	47.9%	12	25.0%	13	27.1%	Chi:1.258 (p:0.533)
	Female	24	40.7%	13	22.0%	22	37.3%	
Age	<60 years	24	40.7%	16	27.1%	19	32.2%	Chi:1.119 (p:0.571)
	> = 60 years	23	47.9%	9	18.8%	16	33.3%	
Marital status	Single	15	34.9%	10	23.3%	18	41.9%	Chi:3.643 (p:0.457)
	Married	24	51.1%	10	21.3%	13	27.7%	
	Divorced-Widowed	8	47.1%	5	29.4%	4	23.5%	
Employment status	Working	14	37.8%	10	27.0%	13	35.1%	Chi:3.963 (p:0.682)
	Retired	17	53.1%	7	21.9%	8	25.0%	
	Unemployed	13	40.6%	8	25.0%	11	34.4%	
	Housewife	3	50.0%	0	0.0%	3	50.0%	
Educational level	Illiterate	0	0.0%	2	66.7%	1	33.3%	Chi:6.822 (p:0.556)
	Primary	14	48.3%	6	20.7%	9	31.0%	
	Secondary	16	48.5%	6	18.2%	11	33.3%	
	Technical	4	50.0%	3	37.5%	1	12.5%	
	Higher	13	38.2%	8	23.5%	13	38.2%	

		Depression						
		No symptoms		Minor symptoms		Moderate - severe		
		N°	%	N°	%	N°	%	
Sex	Male	10	20.8%	14	29.2%	24	50.0%	Chi:4.914 (p:0.086)
	Female	23	39.0%	17	28.8%	19	32.2%	
Age	<60 years	19	32.2%	20	33.9%	20	33.9%	Chi:2.475 (p:0.290)
	> = 60 years	14	29.2%	11	22.9%	23	47.9%	
Marital status	Single	8	18.6%	16	37.2%	19	44.2%	Chi:9.050 (p:0.060)
	Married	20	42.6%	8	17.0%	19	40.4%	
	Divorced -Widowed	5	29.4%	7	41.2%	5	29.4%	
Employment status	Working	9	24.3%	18	48.6%	10	27.0%	Chi:15.999 **(p:0.014)**
	Retired	9	28.1%	9	28.1%	14	43.8%	
	Unemployed	11	34.4%	4	12.5%	17	53.1%	
	Housewife	4	66.7%	0	0.0%	2	33.3%	
Educational level	Illiterate	0	0.0%	2	66.7%	1	33.3%	Chi:11.714 (p:0.164)
	Primary	6	20.7%	11	37.9%	12	41.4%	
	Secondary	11	33.3%	7	21.2%	15	45.5%	
	Technical	5	62.5%	3	29.2%	0	0.0%	
	Higher	11	32.4%	8	28.8%	15	44.1%	

In the case of depression, 50% of men and 32.2% of women showed moderate to severe symptoms. Patients aged over 60 whose marital status was ‘single’ or ‘married’ mostly presented with moderate to severe symptoms. According to educational level, 66.7% of illiterate patients showed mild symptoms, while moderate and severe symptoms predominated in patients with primary and secondary education. In terms of employment status, 48.6% of active workers showed mild symptoms, while moderate and severe symptoms predominated in retirees and the unemployed (sig < 0.05).

In Table 3, thirty-nine percent of patients with sleep disorders had moderate to severe anxiety symptoms, and among those without sleep disorders, 25% also experienced symptoms of that severity. Regarding the number of medications, 55.6% of those who consume more than six medications show severe symptoms of anxiety. However, no statistically significant association was found (p > 0.05).

**Table 3.** Depression and anxiety and their association with sleep disorder variables and medication quantity.

		Anxiety						
		No symptoms		Minor symptoms		Moderate - severe		
		N°	%	N°	%	N°	%	
Sleep disorders	Yes	24	40.7%	12	20.3%	23	39.0%	Chi:2.413 (p:0.299)
	No	23	47.9%	13	27.1%	12	25.0%	
N° of medicines	1–3	20	46.5%	8	18.6%	15	34.9%	Chi:4.059 (p:0.398)
	4–6	24	43.6%	16	29.1%	15	27.3%	
	> 6	3	33.3%	1	11.1%	5	55.6%	

		Depression						
		No symptoms		Minor symptoms		Moderate - severe		
		N°	%	N°	%	N°	%	
Sleep disorders	Yes	15	25.4%	17	28.8%	27	45.8%	Chi:2.270 (p:0.321)
	No	18	37.5%	14	29.2%	16	33.3%	
N° of medicines	1–3	10	23.3%	13	30.2%	20	46.5%	Chi:3.577 (p:0.466)
	4–6	19	34.5%	17	30.9%	19	34.5%	
	> 6	4	44.4%	1	11.1%	4	44.4%	

Regarding depression and its relationship with the variables described above, it was found that 45.8% of those with sleep disorders showed moderate to severe symptoms of depression and 28.8% showed mild symptoms. Regarding the amount of medication, 46.5% of those who took 1 to 3 medications or more than 6 medications showed moderate to severe symptoms of depression; with no evidence of association (p > 0.05).

In Table 4, about the variables age and duration of dialysis, there were no differences among the study groups. However, significant differences were observed in several biochemical parameters, such as albumin (t: 2.767; p:0.003) and hemoglobin values, which are higher in hemodialysis patients (t: 2.326; p = .012), with a moderate effect size (d = 0.504). With regard to urea, the highest levels are found in peritoneal dialysis patients (t:-1.956; p: .027), as are calcium values (t: −1.669; p: .049) and parathyroid hormone levels (t: −2.391; p: .011), with mainly moderate effect sizes, suggesting that the type of dialysis influences the biochemical profile of patients.

**Table 4.** Comparison of laboratory variables according to type of dialysis in patients with end-stage renal disease.

	Type of dialysis	N	Media	Standard deviation	Test t
Age	Haemodialysis	67	55.134	15.43	−1.027 (p: .153)
	Peritoneal dialysis	40	58.325	15.75	d de Cohen (−.205)
Duration of dialysis (years)	Haemodialysis	66	5.106	5.24	−.759 (p: .225)
	Peritoneal dialysis	40	5.950	6.02	d de Cohen(−.152)
Albumin 3,8–5,0 g/dL	Haemodialysis	67	3.951	.48	**2.767 (p: .003)**
	Peritoneal dialysis	40	3.685	.46	**d de Cohen(.553)**
Urea 12–54 mg/dL	Haemodialysis	67	128.247	45.77	**−1.956 (p: .027)**
	Peritoneal dialysis	40	165.050	142.54	**d de Cohen(−.391)**
Hemoglobin >12 g/dL (Women) >13 g/dL (Men)	Haemodialysis	67	10.494	1.11	**2.326 (p: .012)**
	Peritoneal dialysis	40	9.842	1.55	**d de Cohen(.504)**
Serum calcium 8,5–10,5 mg/dL	Haemodialysis	67	8.441	1.67	**−1.669 (p: .049)**
	Peritoneal dialysis	40	9.036	1.97	**d de Cohen(−.334)**
Phosphorus 2,5–4,5 mg/dL	Haemodialysis	67	4.989	1.69	−1.594 (p: .057)
	Peritoneal dialysis	40	5.544	1.83	d de Cohen(−.318)
Parathyroid hormone 10–55 pg/mL	Haemodialysis	67	201.292	149.96	**−2.391 (p: .011)**
	Peritoneal dialysis	40	514.895	821.50	**d de Cohen(−.609)**

Table 5: In relation to anxiety, only hemoglobin showed a statistically inverse correlation (rho = −0.197; p = 0.042), indicating that lower hemoglobin levels were associated with higher anxiety levels. No significant relationship was observed among anxiety and dialysis duration, albumin, urea, calcium, phosphorus, or parathyroid hormone (p > 0.05).

**Table 5.** Laboratory variables in dialysis patients and their association with depression and anxiety.

			Anxiety	Depression
Spearman’s rho	Duration of dialysis (years)	Correlation coefficient	−0.061	−0.048
		Sig. (bilateral)	0.533	0.627
		N	106	107
	Albumin 3,8–5,0 g/dL	Correlation coefficient	−0.080	−0.053
		Sig. (bilateral)	0.413	0.590
		N	107	107
	Urea 12–54 mg/dL	Correlation coefficient	−0.082	**−.204** ^*^
		Sig. (bilateral)	0.402	0.036
		N	107	107
	Hemoglobin >12 g/dL (Women) >13 g/dL (Men)	Correlation coefficient	**−.197** ^*^	**−.199** ^*^
		Sig. (bilateral)	0.042	0.041
		N	107	107
	Calcium 8,5–10,5 mg/dL	Correlation coefficient	−0.137	−0.172
		Sig. (bilateral)	0.159	0.078
		N	107	107
	Phosphorus 2,5–4,5 mg/dL	Correlation coefficient	−0.053	**−.228** ^*^
		Sig. (bilateral)	0.590	0.019
		N	107	107
	Parathyroid hormone 10–55 pg/mL	Correlation coefficient	0.028	−0.039
		Sig. (bilateral)	0.776	0.691
		N	107	107

About depression, a relationship was identified with urea (rho = −0.204; p = 0.036), hemoglobin (rho = −0.199; p = 0.041), and phosphorus (rho = −0.228; p = 0.019), indicating that when hemoglobin and phosphorus levels decrease, depression levels increase. The duration of dialysis, albumin, calcium, and parathyroid hormone did not show association with depression (p > 0.05).

Table 6: The multivariate ordinal regression model indicates that anxiety in dialysis patients is mainly associated with older age, lower calcium levels, and single marital status. In contrast, the remaining biochemical parameters, comorbidities, gender, educational level, and drug treatment do not show a significant association with anxiety.

**Table 6.** Multivariate ordinal regression analysis to estimate factors associated with anxiety.

		Estimate	Std. Error	Wald	df	Sig.	95% Confidence interval	
							Lower bound	Lower bound
Threshold	[Anxiety = 0]	−6.182	4.079	2.296	1	.130	−14.176	1.813
	[Anxiety = Mild]	−4.850	4.064	1.424	1	.233	−12.815	3.115
Location	Age	.064	.027	5.559	1	.018	.011	.118
	Duration of dialysis	.011	.051	.043	1	.836	−.089	.110
	Albumin	−.065	.582	.013	1	.911	−1.205	1.075
	Urea	−.007	.006	1.118	1	.290	−.019	.006
	Hemoglobin	−.305	.196	2.429	1	.119	−.688	.079
	Calcium	−.554	.265	4.365	1	.037	−1.074	−.034
	Phosphorus	.056	.186	.090	1	.765	−.309	.420
	Parathyroid hormone	.001	.001	.772	1	.380	−.001	.003
	[Sex = Male]	−.466	.486	.917	1	.338	−1.419	.488
	[Sex = Female]	0 ^a^	.	.	0	.	.	.
	[Marital status = Single]	1.845	.832	4.923	1	.027	.215	3.475
	[Marital status = Married]	.589	.778	.573	1	.449	−.936	2.115
	[Marital status = divorced/widowed]	0 ^a^	.	.	0	.	.	.
	[Employment statusl = Active]	−.374	1.146	.107	1	.744	−2.621	1.872
	[Employment status = Retired]	−2.173	1.213	3.210	1	.073	−4.551	.204
	[Employment status = Unemployed.]	−.671	1.259	.284	1	.594	−3.139	1.798
	[Employment status = Housewife]	0 ^a^	.	.	0	.	.	.
	Educational level [Illiterate]	1.697	1.885	.811	1	.368	−1.998	5.392
	[Educational level = Primary]	−.506	.701	.522	1	.470	−1.880	.867
	[Educational level = Secondary]	−.553	.619	.799	1	.371	−1.765	.660
	[Educational level = Technical]	−.584	1.143	.261	1	.609	−2.824	1.656
	[Educational level = Higher]	0 ^a^	.	.	0	.	.	.
	[Primary cause ERC = T2D]	1.444	1.570	.846	1	.358	−1.634	4.522
	[Primary cause ERC=Hypertension]	.453	1.562	.084	1	.772	−2.609	3.514
	[Primary cause ERC = Nephritis]	.799	3.062	.068	1	.794	−5.203	6.802
	[Primary cause ERC = Glomerulonefritis]	.655	1.687	.151	1	.698	−2.651	3.961
	[Primary cause ERC = Unknown]	.209	1.604	.017	1	.897	−2.935	3.353
	[Primary cause ERC = Another]	0 ^a^	.	.	0	.	.	.
[Comorb = T2D]	−2.146	1.448	2.196	1	.138	−4.985	.692
[Comorb = Cardiac disease]	−1.001	.979	1.045	1	.307	−2.919	.918
[Comorb = Cancer]	−1.479	1.171	1.596	1	.206	−3.774	.815
[Comorb = Vascular disease]	23.965	.000	.	1	.	23.965	23.965
[Comorb = Hepat C]	−.890	.861	1.068	1	.301	−2.578	.798
[Comorb = 0]	0 ^a^	.	.	0	.	.	.
[Sleep disorder = Yes]	.774	.499	2.406	1	.121	−.204	1.752
[Sleep disorder = No]	0 ^a^	.	.	0	.	.	.
[Medicines = 1 a 3]	−.804	.939	.733	1	.392	−2.645	1.036
[Medicines = 4 a 6]	−.855	.884	.935	1	.334	−2.587	.878
[Medicines >6]	0 ^a^	.	.	0	.	.	.

Liaison function: Logit.

Nagelkerke’s R: .433

^a^ This parameter is set to zero because it is redundant.

Table 7: The ordinal logistic regression model shows that hemoglobin is mild associated with the level of depression (B = −0.404; p = .046). Likewise, the presence of sleep disorders increased the probability of higher levels of depression (B = 1.058; p = .026). The model shows adequate fit and explains 43.3% of the variability (Nagelkerke’s R ^2^ = .433); however, the values are not statistically significant when the Bonferroni correction procedure is applied.

**Table 7.** Multivariate ordinal regression analysis to estimate factors associated with depression.

		Estimate	Std. Error	Wald	df	Sig.	95% Confidence interval	
							Lower bound	Upper bound
Threshold	[Depression = 0]	−3.853	3.194	1.456	1	.228	−10.113	2.406
	[Depression = Mild]	−2.048	3.178	.415	1	.519	−8.277	4.182
Location	Age	.034	.025	1.826	1	.177	−.015	.082
	Duration of dialysis	−.055	.046	1.421	1	.233	−.146	.036
	Albumin	−.790	.563	1.969	1	.161	−1.894	.314
	Urea	.003	.003	.811	1	.368	−.004	.010
	Hemoglobin	−.404	.202	3.994	1	.046	−.799	−.008
	Calcium	−.224	.186	1.446	1	.229	−.589	.141
	Phosphorus	−.087	.193	.201	1	.654	−.465	.292
	Parathyroid hormone	.000	.001	.147	1	.701	−.001	.002
	[Sex = Male]	.847	.475	3.177	1	.075	−.084	1.777
	[Sex = Female]	0a	.	.	0	.	.	.
	[Marital status = Single]	.801	.764	1.100	1	.294	−.696	2.298
	[Marital status = Married]	.163	.714	.052	1	.819	−1.237	1.563
	[Marital status = Divorced/widowed]	0a	.	.	0	.	.	.
	[Employment status = Active]	1.725	1.318	1.714	1	.191	−.858	4.309
	[Employment status = Retired]	1.400	1.396	1.006	1	.316	−1.336	4.136
[Employment status = Unemployed.]	2.316	1.413	2.686	1	.101	−.454	5.086
[Employment status = Housewife]	0a	.	.	0	.	.	.
[Educational level = Illiterate]	1.637	1.744	.880	1	.348	−1.782	5.055
[Educational level = Primary]	.151	.683	.049	1	.825	−1.187	1.489
[Educational level = Secondary]	−.362	.596	.369	1	.544	−1.530	.806
[Educational level = Technical]	−2.219	1.161	3.652	1	.056	−4.495	.057
[N_Educa = Higher]	0a	.	.	0	.	.	.
[Primary cause ERC = T2D]	.655	1.429	.210	1	.647	−2.145	3.455
[Primary cause ERC=Hypertension]	−.326	1.431	.052	1	.820	−3.130	2.478
[Primary cause ERC = Nephritis]	−.351	2.345	.022	1	.881	−4.947	4.245
[Primary cause ERC = Glomerulonephritis]	.537	1.564	.118	1	.731	−2.529	3.602
[Primary cause ERC=Unknown]	−.742	1.500	.244	1	.621	−3.681	2.198
[Primary cause ERC = Another]	0a	.	.	0	.	.	.
[Comorb = T2D]	1.869	1.226	2.321	1	.128	−.535	4.272
[Comorb = Cardiac disease]	1.328	.873	2.315	1	.128	−.383	3.040
[Comorb = Cancer]	1.578	1.115	2.003	1	.157	−.607	3.763
[Comorb = Vascular disease]	1.422	2.027	.492	1	.483	−2.551	5.395
[Comorb = Hepat C]	1.245	.812	2.355	1	.125	−.345	2.836
[Comorb = None]	0a	.	.	0	.	.	.
[Sleep disorders = Si]	1.058	.475	4.960	1	.026	.127	1.989
[Sleep disorders = No]	0a	.	.	0	.	.	.
[Medicines =1 a 3]	.768	.942	.666	1	.415	−1.077	2.614
Medicines =4 a 6]	.820	.893	.842	1	.359	−.931	2.570
[Medicines >6]	0 ^a^	.	.	0	.	.	.

Liaison function: Logit.

Nagelkerke’s R: .433

^a^ This parameter is set to zero because it is redundant.

## IV. Discussion

A total of 107 patients with stage G5 chronic kidney disease (CKD) undergoing dialysis during the study period were studied; 67 were on hemodialysis (48% men and 52% women) and 40 were on peritoneal dialysis (40% men and 60% women). The average age of the patients was 55.134 ± 15.43 and 58.325 ± 15.75 years, respectively.

CKD is a health problem that negatively affects patients’ quality of life and is associated with various psychiatric conditions. ^12^ It is believed that these patients live with chronic stress that increases with the severity of kidney damage. ^3^ On the other hand, dietary restrictions, associated comorbidities, adverse effects of medication, changes in self-perception, and fear of dying are more pronounced in the final stages of CKD. ^13^ Therefore, hemodialysis patients are at greater risk for mental illness, mood changes, and functional disabilities, among other changes. ^12– 15^

Mental health problems such as anxiety, ^1– 3, 6, 11, 15, 20^ depression ^1, 2, 9– 11, 20^ and sleep disorders ^3, 5, 14^ in patients with G5 CKD have been widely described in a series of studies that include demographic data such as age, marital status, employment status, educational level, and clinical data on patients. One study report that among hemodialysis patients, there was a predominance of patients aged 59.1 ± 16, male, married, illiterate, and unemployed, ^1^ and another study found that 72.31% of patients with CKD were married or had a partner. ^34^ In this study, females predominated in both dialysis groups, with a higher proportion in peritoneal dialysis patients. Hemodialysis patients were mostly single, unemployed, and had primary and secondary education, while peritoneal dialysis patients were predominantly married, employed, and had higher education.

Regarding the primary causes of end-stage renal disease (ESRD), studies report diabetes, ^1, 35^ glomerulonephritis, ^35^ hypertension, ^1, 35^ and vascular nephropathy. ^35^ In 25.6% of cases, the primary cause is unknown. ^1^ Diabetes mellitus and hypertension share common pathophysiological mechanisms for the development and progression of ESRD ^36, 37^ and it has been described that the microvascular complications characteristic of DM are largely responsible for the progression to stage 5 ESRD requiring dialysis or kidney transplant ^38, 39^; It is also noted that high blood pressure is both a cause and a consequence of CKD. ^40^ In the present study, the main primary causes of end-stage CKD, in order of frequency, are hypertension and type 2 diabetes mellitus, regardless of the type of dialysis. The primary cause of CKD was unknown in 15% of patients on hemodialysis and 12.5% of patients on peritoneal dialysis; these values are lower than those reported in other studies, which indicate that the primary cause of CKD is unknown in up to 25.6% of cases. ^1^

Diabetes mellitus and hypertension are reported among the most frequent comorbidities of CKD ^35, 41^ as well as a history of cardiovascular disease. ^35^ In the present study, heart disease/hypertension and sleep disorders predominated in the peritoneal dialysis group, while in the hemodialysis group, more than 56% showed no comorbidities and, to a lesser extent, heart disease and peripheral vascular disease were present. About sleep disorders, one study found that insomnia is associated with comorbidities, but also with advanced age, fatigue, and pruritus. ^42^ Likewise, peritoneal dialysis patients had a higher drug burden, with a predominance of 4 to 6 medications, while hemodialysis patients more frequently used 1 to 3 drugs.

It has been reported that one in five patients with CKD experience depression or anxiety when starting dialysis. ^1^ In a cross-sectional study in Morocco, the prevalence of anxiety was 25.2% to 69.3% and for depression it was 34% to 67% in patients on hemodialysis. ^35^ Another Moroccan study reported a prevalence of anxiety of 28.2% and depression of 23.1% in patients receiving the same treatment for CKD. ^9^ In Peru, studies have identified depression in hemodialysis patients, with a predominance of mild depression in 48.9%. ^43^ In the present study, the prevalence of anxiety symptoms was 56% and for depressive symptoms 69.15%, regardless of the type of dialysis. In all these studies, the HADS ^21– 23^ questionnaire was used to detect anxiety and depression symptoms and assess their severity in dialysis patients ^22, 23^; it is more specific than other tests because it excludes possible physical pathologies that are often somatized from its responses. ^20^

When using the HADS, the mean anxiety score was 7.7 ± 4.6 (range: 0–19) and the mean depression score was 7.4 ± 4.2 (range: 0–15) in hemodialysis patients. ^1^ In the present study, the mean score for anxiety was 7.985 ± 4.675 and for symptoms of depression 9.833 ± 3.431 in the same type of patients; similar results for anxiety symptoms and higher frequency for depressive symptoms. Among patients on peritoneal dialysis, the mean score for anxiety was 8.725 ± 4.723 and for symptoms of depression 8.325 ± 4.208. These findings differ from a study conducted in India using the same instrument in patients on hemodialysis. The anxiety score was reported as 13.82 ± 2.73 and for depression as 12.58 ± 3.12, which the authors consider to be attributable to the prevailing conditions in the Indian population, such as low socioeconomic status, poor or inadequate access to health services, and low educational level, which are the main factors contributing to anxiety, stress, or depression in dialysis patients in that country. ^3^

When analyzing by type of dialysis, symptoms of depression predominated over anxiety among hemodialysis patients (with a higher proportion of moderate to severe depression symptoms at 41.8%); whereas among participants undergoing peritoneal dialysis moderate to severe symptoms of anxiety were more frequent. These results are like those reported in other studies, which indicate that up to 74% of hemodialysis patients suffer from depression in any of its forms ^9^ and that this pathology is significantly more frequent among individuals undergoing chronic hemodialysis compared to peritoneal dialysis (30.6% versus 20.4%; p = 0.04). ^8^ Another study reported that 41.79% of patients with advanced CKD presented anxiety symptoms, with 13.43% having a clinical diagnosis of anxiety; however, they defined advanced CKD as stage G3 and above, unlike the present study, which only includes patients with stage G5 CKD on dialysis, implying greater vulnerability and risk. Similarly, in the case of depression, they reported 25.38% of depression ^34^; unlike the present study, in which 41.8% of patients on hemodialysis present moderate and severe depressive symptoms. Another study reported that both anxiety and depression were prevalent in patients on peritoneal dialysis, a significant number of whom had previously been on hemodialysis and switched to peritoneal dialysis due to venous access exhaustion, an experience that could have negatively affected their perception of the disease. ^45^ The fact that peritoneal dialysis is performed at home should also be considered, as being away from a healthcare facility could increase anxiety due to fear of complications during the process, whereas hemodialysis, being performed in a healthcare facility, could give patients a feeling of having medical support close at hand. Additional research is required to examine the perceptions contributing to mental health issues related to dialysis.

When analyzing the association between demographic variables such as gender, age, marital status, employment status, and educational level and symptoms of anxiety, it was found that more than 50% of women and men presented some symptoms of anxiety. Regarding the severity of symptoms, 37.3% of women and single people presented moderate to severe symptoms of anxiety, while mild symptoms predominated among illiterate people. However, no statistically association (sig > 0.05) was found between these variables and anxiety symptoms, unlike another study that reports a relationship between age and sleep disorders and anxiety, ^1^ and between educational level and higher levels of depression and anxiety. ^43^

Likewise, 61% of women and 79% of men had some degree of depressive symptoms. Fifty percent of men, one-third of women, those over 60 years of age, single people, patients with primary and secondary education, retirees, and unemployed people presented moderate to severe symptoms. Among married people, 40.4% presented moderate to severe symptoms, while most illiterate and active workers showed mild symptoms. In this regard, a Peruvian study reports a greater tendency toward moderate depression in older adult, widowers, followed by divorcees and singles; statistically significant findings that could be related to the absence of a partner and lack of social support to manage the disease and attend dialysis sessions. ^43^

This study found a mild association between employment status and symptoms of depression (p < 0.05). Other studies have also found a correlation between unemployment and depression ^45, 46^; between advanced age, ^1, 43^ low educational level, ^1, 10, 43, 47^ retirement, poor financial situation, and depression. ^45^ Unemployment is a risk factor for depression because it involves stressors related to economic autonomy and makes patients more vulnerable to disease and dialysis treatment. ^43^

Other studies report a correlation between marital status, educational level, ^1, 43^ and socioeconomic status with depression ^1^; data that differ from our results It has been reported that renal patients with lower educational levels have a greater tendency toward depression than those with other educational levels. ^43^

A systematic review of 28 studies showed that depression and anxiety are highly prevalent among patients with CKD on dialysis ^44^; coinciding with the high frequency of these conditions reported in the present study. As already mentioned, anxiety and depression are related to having CKD, ^3^ even more so if dialysis is required; therefore, it is important to consider other psychosocial factors linked to anxiety and depression, such as family support and coping styles. ^44^

Sleep disorders are among the factors that affect the quality of life of patients with CKD, and their prevalence ranges from 41% to 83% depending on the instrument used for their assessment. They can manifest as difficulty falling asleep, difficulty staying asleep, daytime sleepiness, and chronic fatigue. ^3^ In the present study, 39% and 45.8% of patients with this diagnosis present moderate to severe symptoms of anxiety and depression, respectively, and the presence of sleep disorders may increase anxiety levels; (B = 1.058; p = 0.026), according to the correlation model used, which explains 43.3% of the variability (Nagelkerke’s R ^2^ = 0.433); however, the Bonferroni correction shows that there is no significant association (sig > 0.003). Another study only reports sleep disorders as factors associated with anxiety. ^1^

In another study, significantly high levels of insomnia were found in people over 60, divorced or widowed (p = 0.007), in those with comorbidity (p = 0.001), with fatigue after hemodialysis (p = 0.001), with continuous fatigue (p = 0.001), and in those who had pruritus (p = 0.007) and joint stiffness (p = 0.001). ^42^ An association has also been reported among anxiety, depression, and sleep quality in older adults with albuminuria and decreased glomerular filtration rate, although the mechanism underlying this association is unclear and could be due to changes in hormone levels associated with CKD. ^48^ Sleep disorders also occur due to changes in corticothalamic activity, with elevated concentrations of adrenocorticotropic hormone and cortisol, which make it difficult to fall asleep or stay asleep. Therefore, sleep quality is directly related to anxiety and depression. ^49, 50^

Among the patients with moderate to severe anxiety symptoms, polypharmacy (>6 medications) was predominant. However, there was no statistical association between this variable and symptoms of anxiety and depression (p > 0.05), unlike another study in which anxiety and depression scores were significantly correlated with the number of medications (P = 0.022 and P = 0.003, respectively). ^1^ Polypharmacy is reported to be particularly common in older adults with CKD and carries the risk of adverse effects, cumulative toxicity, drug interactions, and functional decline. ^51^

It has been reported that factors related to dialysis, such as the type of treatment, travel time, waiting hours, and place of treatment, dietary restrictions, lack of family or social support, and unemployment can cause emotional disorders and, therefore, higher levels of depression and anxiety. Likewise, older age and longer time on dialysis tend to increase depression ^43^; unlike the present study, in which the demographic variables age and duration of dialysis show no differences between the groups studied and do not correlate with anxiety or depression.

In relation to the biochemical parameters studied, higher levels of albumin (t: 2.767; p: 0.003) and hemoglobin  (t: 2.326; p = .012) were observed, with a moderate effect size (d = 0.504); in hemodialysis patients, with statistically significant differences, while urea values (t: −1.956; p: .027); calcium (t: −1.669; p: .049) and parathyroid hormone (t: −2.391; p: .011) were higher in patients on peritoneal dialysis (mainly moderate effect size), suggesting that the type of dialysis influences the biochemical profile of patients. Some studies report the prevalence of hyperphosphatemia in dialysis patients, ^52^ which could be related to poor phosphate binding to chelators and the complexity of dietary management; aspects that are often linked to depression. ^44^ In the present study, phosphorus levels were adequate (4.989 mg/dL for hemodialysis and 5.544 mg/dL for peritoneal dialysis); these data are like those of a study in which 68.2% of patients with end-stage CKD had phosphorus levels ≥5.5 mg/dL. ^44^

In relation to biochemical parameters and anxiety, only hemoglobin showed a mild inverse correlation (rho = −0.197; p = 0.042). Another study reported a significant relationship between low hematocrit levels and anxiety (p < 0.05) and low hematocrit levels (p < 0.01) and hemoglobin (p < 0.05) in patients with depression. ^53^ However, low hemoglobin levels caused by insufficient erythropoietin production are common in CKD, and their symptoms could be confused with those of depression. ^54^

No significant relationship was observed between anxiety and dialysis duration, albumin, urea, calcium, phosphorus, or parathyroid hormone (p > 0.05), data like another study in terms of the variable dialysis duration, serum calcium, and phosphorus, which showed no association with depression and anxiety. ^52^ Another study reported that patients with generalized anxiety disorder and elevated parathyroid hormone levels have poor sleep quality and higher levels of anxiety. They observed a strong relationship between peripheral biomarkers of calcium homeostasis imbalance, insomnia, poor sleep quality, and anxiety symptoms. ^55^

In the present study, a relationship was identified between depression and serum urea levels (rho = −0.204; p = 0.036), hemoglobin (rho = −0.199; p = 0.041), and serum phosphorus (rho = −0.228; p = 0.019). A significant association has been reported in depression and the glomerular filtration rate (GFR), serum urea, and hemoglobin, as well as in diabetes, serum urea, and anxiety. ^35^ Another study found that anxiety and depression scores have a strong negative correlation with GFR, hemoglobin, and serum calcium (p < 0.01) and a positive correlation with total white blood cell count, urea, creatinine, and phosphate (p < 0.05). ^3^ Likewise, in a longitudinal study of more than 2,000 patients with CKD on hemodialysis and diagnosed with depression, it was found that serum urea levels were more strongly associated with depressive symptoms than with depressive illness, although this was not statistically significant. ^56^ Elevated serum urea levels in end-stage CKD are associated with accelerated atherosclerosis. Therefore, brain aging and associated pathologies such as depression and neurocognitive disorders are increased. Likewise, the carbamylation process caused by isocyanate or carbamyl phosphate leads to urea accumulation, which has an impact on oxidative stress, apoptosis, and synaptic plasticity. ^55, 56^

The association between CKD and the high prevalence of cerebrovascular disease, cognitive impairment, and depression has also been described ^7, 56^; as well as the role of uremic toxins in the development of some neurological disorders, as they have direct neurotoxic actions such as astrocyte activation and neuronal death, and indirect actions via vascular effects such as endothelial dysfunction, calcification, ^7^ and inflammation. ^7, 57, 58^ Among these toxins is indoxyl sulfate, which has proven neurotoxic effects. ^7^ On the other hand, CKD is a complex pathology characterized by chronic inflammation, oxidative stress, and vascular damage, which, combined with dysregulation of the hypothalamic-pituitary-adrenal axis, is involved in the pathogenesis of depression. ^58^

Regarding hemoglobin and phosphorus levels, this study found a inverse relationship between these variables and higher levels of depressive symptoms. As already mentioned, anemia is common in CKD patients on dialysis and can be caused by reduced erythropoietin production, erythropoietic depressants induced by uremic toxins, iron deficiency, and a decrease in the mean life span of erythrocytes. Anemia is significantly associated with depression and, together with albumin levels, is among the risk factors for depression in older patients with CKD. ^50^

Another study reports a significant correlation between nutritional status assessed by serum albumin and depressive symptoms in patients with CKD, ^59^ unlike the present study, which found no association between serum albumin levels and symptoms of anxiety or depression. With regard to phosphorus, it is reported that elevated levels could be related to poor adherence to phosphate binders and the complexity of dietary management, aspects that are often linked to depression. ^44^

No statistically significant association was found in the duration of dialysis, albumin, calcium, parathyroid hormone, and depression (p > 0.05). Multivariate ordinal regression analysis to estimate factors associated with anxiety and depression demonstrated a mild association among anxiety and older patient age, lower calcium levels, and single marital status; hemoglobin was inversely associated with depression (B = −0.404; p = .046). Other studies found that age showed a statistically significant inverse and weak relationship with depression and that women had higher levels of anxiety and depression than men ^60^; that females had higher levels of depression, anxiety, and stress and experienced decreases in albumin, calcium, and vitamin D, concluding that sex influences the relationship between inflammation, nutrition, and psychological distress in hemodialysis patients. ^61^

Depression also has behavioral consequences that can negatively affect medical outcomes. In patients with CKD on dialysis, depression has been associated with non-compliance with drug treatment, dietary non-compliance, weight gain between dialysis sessions, and non-attendance at dialysis sessions. ^57^ Therefore, we believe that care for patients with CKD on dialysis must go beyond the biomedical model to improve clinical outcomes and treatment adherence. This requires addressing anxiety through lifestyle changes, cognitive behavioral therapy, regular exercise, and relaxation and mindfulness activities. ^1, 44, 62^

Current research reports some factors that cause depression and mentions the bidirectional relationship between CKD and depression ^57^; However, we believe that further study of the mechanisms of depression and the biochemical markers involved is required, as well as the implementation of protocols for evaluation at least twice a year for the timely detection of these health problems through self-administered questionnaires followed by psychological or psychiatric consultation. It is also necessary to propose improvements in the institution aimed at providing organizational, family, and personal resources to deal with anxiety and depression, including information and education about dialysis.

### Limitations of the study

This study was subject to the limitations inherent in any cross-sectional study, which does not allow for the establishment of causality and is vulnerable to selection bias; consequently, this study presents a baseline picture, making necessary to follow up on the cohort, including analysis of polypharmacy, treatment received for anxiety or depression, and family and social support. A longitudinal research design is required to confirm many of the findings of this study. Likewise, the statistical reliability of the findings in the logistic regression analysis, the small sample size and the low number of variables per participant are further limitations.

Limitations were also encountered in the analysis of biochemical markers; although these had been included in the protocol for patients on dyalisis, certain logistical problems at the hospital delayed the collection and evaluation of the clinical tests.

### Scientific contribution

The study provides primary data on the clinical, laboratory and demographic factors associated with anxiety and depression in Peruvian patients with chronic kidney disease who are on dyalisis. In Peru, the literature on these topics remains limited; therefore, the results will shed light on the psychosocial issues facing the study population which will enable the formulation of proposals for institutional improvements aimed at a comprehensive and interdisciplinary approach to the care of these patients through updated protocols.

## Conclusions

Results show that symptoms of anxiety and depression are common problems amongst dialysis patients, with variations depending on sociodemographic and clinical factors, as well as the type of dialysis treatment. However, the results should be interpreted with caution due to the sample size, which limits generalization to other populations, and the correlational design, which does not allow definitive causal relationships to be established.

The diagnosis and commencement of dialysis for CKD have a profound emotional impact, forcing patients to drastically alter their lifestyle. This transition often triggers anxiety, depression and chronic stress; psychological states that cause physiological changes — such as elevated cortisol levels and high blood pressure — which accelerate kidney damage. Furthermore, emotional distress compromises adherence to treatment and limits social participation due to a loss of autonomy. Consequently, given that psychological reactions directly influence disease progression and mortality, a multidisciplinary approach is essential. Integrating psychological care from the early stages is key to developing active coping strategies, optimising mental health and, consequently, safeguarding the patient’s quality of life.

## Data Availability

Zenodo. Prevalence and factors associated with depression and anxiety in patients with chronic kidney disease. Available at https://doi.org/10.5281/zenodo.18968127. ^61^ This project contains the following underlying data:
1. Data collection form 2. Hospital Anxiety and Depression Scale 3. DATABASE ok Anxiety and Depression in ERC.xlsx 4. Informed consent English Data is available under the terms of the Creative Commons Attribution 4.0 International license
