# Prevalence and Factors Associated with Depression and Anxiety in Patients with Chronic Kidney Disease

**DOI:** 10.12688/f1000research.178880.3

**Published:** 2026-08-08

**Authors:** Evelyn Goicochea Rios, Ana Chian Garcia, Jhony Paredes Silva, Jessica Vicuña Villacorta

**Affiliations:** 1 La Libertad, Universidad Cesar Vallejo Facultad de Ciencias Medicas, Trujillo, La Libertad, 13001, Peru

**Keywords:** Anxiety, depression, hemodyalisis, peritoneal dyalisis, uremic toxins, HEADS, diabetes mellitus, hypertension

## Abstract

**Background:**

Depression and anxiety are common in chronic kidney disease (CKD) and can increase morbidity and mortality. Therefore, the objective was to analyze their prevalence and the associated demographic, clinical, and laboratory factors in CKD patients undergoing dialysis.

**Methodology:**

Correlational design research with prospective cohort. Sample of 107 patients, 67 on hemodialysis and 40 on peritoneal dialysis (PD). The Hospital Anxiety and Depression Scale questionnaire was used after obtaining free and informed consent. The Spearman correlation coefficient, Chi ^2^ and multivariate ordinal logistic regression analysis were used.

**Results:**

Females predominated in both dialysis groups. Hemodialysis patients were mostly single, unemployed, and had primary or secondary education, whereas peritoneal dialysis patients were predominantly married, employed, and had higher education. Hypertension and diabetes mellitus were the leading causes of CKD. The overall prevalence of anxiety and depressive symptoms was 56.0% and 69.2%, respectively. Depression was more frequent among hemodialysis patients, whereas moderate-to-severe anxiety symptoms predominated in the peritoneal dialysis group. Depression was significantly associated with employment status, serum urea, hemoglobin, and phosphorus levels, while anxiety was inversely associated with hemoglobin levels. Multivariable ordinal logistic regression identified older age and lower serum calcium levels as independent correlates of greater anxiety severity. Older age, single marital status, sleep disorders, cancer, hepatitis C, and lower serum calcium levels were independently associated with greater depressive symptom severity.

**Conclusion:**

Early identification of anxiety and depressive symptoms in patients with CKD undergoing dialysis is essential to facilitate timely and appropriate management.

## I. Introduction

Depression and anxiety disorders are prevalent in people with chronic kidney disease (CKD), especially in advanced stages and on dialysis. ^1^ It is estimated that depression affects 25% and anxiety 23% of dialysis patients and according to other studies, the prevalence can range from 27 to 30% ^2^ and from 69% to 71% respectively. ^3^

Depression is defined as feelings of sadness, guilt, disinterest, low self-esteem, insomnia, hyporexia/anorexia, fatigue, and lack of concentration and pleasure. ^4^ It has personal repercussions at the psychological, emotional, and behavioral levels, ^2, 5^ and affects treatment compliance, so detecting the factors associated with depression should be a priority. ^3, 5^ Risk factors for depression include being female, low socioeconomic status, stressful life events, lack of social support, serious or chronic illness, and a history of eating disorders. ^5^

Anxiety is characterized by feelings of uncertainty and fear and can manifest as palpitations, tremors, indigestion, nervousness, shortness of breath, and diaphoresis, often being confused with other pathologies. ^6^ However, anxiety disorders have been less studied in patients with advanced stages of CKD, and their impact is largely unknown in this population. ^1, 2^

Chronic kidney disease (CKD) is strongly associated with an increase in mental health problems, including depression and anxiety. ^1– 3^ There is also evidence of its association with central nervous system diseases, cognitive dysfunction, and dementia. People with CKD are more likely to have strokes and subclinical cerebrovascular disease than the general population. It has also been reported that uremic toxins related to kidney damage may predispose individuals to neurological disorders. ^7^

A systematic review covering information on 80,932 individuals with CKD from 27 countries found an overall prevalence of depression of 26.5% and 39.6% when using the International Classification of Diseases. Depression was significantly more common among individuals on chronic hemodialysis compared to pre-dialysis patients (29.9% and 18.5%, respectively). ^8^ Another study of 376 patients with CKD, a third of whom were on hemodialysis, found that 74% suffered from depression, with mild depression predominating (16%). Among the factors significantly associated with depression, they found the duration of CKD since diagnosis, the level of independence in performing routine activities at home, the ability to generate income, and lifestyle changes. ^9^

The association between depression and various sociodemographic variables in patients with CKD has been documented, ^10– 13^ including gender, educational attainment, age group, employment status and duration of CKD ^10^ and monthly income ^13^; other authors consider that family support, ^10, 11^ social support and marital status ^12^ are associated with clinical depression, as are a negative perception of the disease and low self-esteem. ^10^ It is also noted that socio-economic variables affect the quality of life of people with CKD. ^14^

Other studies with CKD patients report that single marital status and female gender ^13, 15^ were significantly associated with depression and anxiety, as well as unemployment and polypharmacy. ^15^ Bivariate and multivariate logistic regression in another study showed that social support and time since CKD diagnosis were significantly associated with depression and anxiety. Other associated factors include uric acid levels ^16^ and family history of mental illness, with patients with CKD and comorbidities being 1.7 times more likely to develop such mental disorders. ^13^

In Peru, most patients with CKD who require dialysis are treated by Essalud and the Integral Health Insurance system. From 2019 to 2023, 205,501 cases of CKD were diagnosed (2.16% of the total insured population), of which 10% were in stage G5 as confirmed by laboratory tests. ^17^ Current treatment modalities do not offer a cure, but they can alleviate symptoms and prolong the life of patients who come to dialysis with great fear because they associate this condition with mortality, radical changes in their lifestyle and personal routine, and uncertainty about the progression of this chronic disease. ^18^ No research on mental illnesses such as depression, anxiety, stress, and sleep disorders in CKD patients on dialysis was found in the Peruvian medical literature. Therefore, the objective of this study is to analyze the prevalence of depression and anxiety in CKD patients and the demographic, clinical, and laboratory factors associated with these pathologies. The findings will provide primary data on this issue in a systematic manner to adapt the protocols for the diagnosis and treatment of depression and anxiety in patients with CKD.

## II. Patients and methods

### Study design, population, and sample

A cross-sectional study was conducted. ^19^ The target population consisted of 160 adults patients diagnosed with CKD on dialysis treated between June and December 2025 at an Essalud Social Security hospital and at the SIS (Seguro Integral de Salud) in the province and district of Trujillo, Peru, who met the following inclusion criteria: signing the free and informed consent form, both sexes, with laboratory results, demographic data, clinical data, and complete records in the medical history. Two patients with incomplete medical records and one minor patient were excluded.

A stratified proportional probability sampling method was used, and the sample size was determined using the formula for finite populations ^19^ with a 95% confidence level and an adjusted error of 5.5%. It was then distributed proportionally in each stratum according to its relative size, comprising 40 patients on peritoneal dialysis and 67 patients on hemodialysis with a diagnosis of stage G5 CKD who met the above inclusion criteria.

### Data collection techniques and instruments

Interviews and document analysis were used. ^19^ Demographic data were recorded on a structured form designed by the researchers: age, sex, marital status, educational level, employment status; clinical data: years on dialysis, primary cause of CKD, weight, height, body mass index (BMI), frequency and type of dialysis, type and number of comorbidities, sleep disturbances, number of medications, and laboratory parameters: hemoglobin, serum albumin, creatinine, urea, blood glucose, ferritin, C-reactive protein, calcium, phosphorus, and parathyroid hormone. This form included the HADS, ^20^ a tool that has been validated in numerous studies as an effective instrument for identifying symptoms of anxiety and depression in diverse populations, including the general population, people with chronic or psychiatric conditions, ^21, 22^ and patients undergoing haemodialysis ^23^ and peritoneal dyalisis. ^20^ Designed to minimise the impact of somatic symptoms, such as headaches, chest discomfort, fatigue or sleep disturbances, it focuses on the emotional and cognitive aspects of anxiety and depression. ^24^ Is characterised by its good internal reliability and test-retest. ^20^ The psychometric properties of the HADS were assessed in a Peruvian study of patients with chronic kidney disease, revealing good internal consistency (overall McDonald’s omega ω = 0.806). The anxiety subscale showed acceptable reliability (ω = 0.757), whilst the depression subscale showed lower consistency (ω = 0.680). ^25^

HADS consists of 14 items related to the emotional and cognitive aspects of depression and anxiety: seven for each subscale. Each item is scored from 0 to 3, and the score ranges from 0 to 21 for each subscale, with a total score ranging from 0 to 42. Scores of 0–7 indicate no significant symptoms, 8–10 indicate mild symptoms, and 11–15 and 16–21 indicate moderate and severe symptoms, respectively. ^21^

The measure was implemented to all patients in the peritoneal dialysis and hemodialysis program. In the case of hemodialysis, patients were interviewed three times a week prior to treatment, depending on availability. In the case of peritoneal dialysis patients receiving home treatment, they were interviewed on the day they attended their outpatient nephrology appointment. The instrument was validated by nephrologists and family doctors. An Aiken V score was obtained, with a value of 0.94. ^26^

### Procedure

The research was authorized by the La Libertad Healthcare Network after review and approval by the Institutional Ethics Committee. The inclusion criteria were then applied to the target population that agreed to participate. Each patient was given an information sheet about the study and, after signing the free and informed consent form, the information from their medical records was collected in an anonymous database. The HADS scale was implemented during the interview. Subsequently, the information was statistically analyzed, and this article was prepared. All variables recorded by the responsible physician were considered, and the results were presented considering the STROBE checklist. ^27^

### Laboratory measurements

The blood samples were taken in accordance with the protocol established for sampling hemodialysis patients (before the start of the treatment session) and, in the case of peritoneal dialysis patients, when they attended the laboratory. The normal ranges for the tests are: serum albumin 3.8–5.0 g/dL; creatinine: women, 0.5–1.1 mg/dL and men, 0.6–1.2 mg/dL; serum urea 12–54 mg/dL ^28^; hemoglobin >12 g/dL for women and > 13 g/dL for men; ferritin 30–400 ng/mL for men and 13–150 ng/dL for women ^29^; serum calcium 8.5–10.5 mg/dL; serum phosphorus 2.5–4.5 mg/dL; parathyroid hormone 10–55 pg/mL; C-reactive protein <1.00 mg/dL and blood glucose 75–110 mg/dL. ^28^ In adults <60 years of age, overweight was defined as a BMI of 25–29.9 and obesity as a BMI >30; while in adults >60 years of age, overweight was considered if BMI 28–31.9 and obesity >32. ^30^

### Data processing

JAMOVI, ^31^ open-access statistical software, was used to record each patient’s data, excluding personal identification details. A database was generated in Excel. Continuous data are presented using means and standard deviation, and categorical data are presented as percentages. ^19^

### Statistical analysis

Descriptive statistics were used to analyze tables, averages, and standard deviations to analyze the behavior of the study variables. For the inferential analysis, the Spearman’s correlation coefficient and Chi ^2^ were used in the case of categorical variables, and multivariate ordinal logistic regression analysis ^32^ was performed to estimate the factors associated with depression and anxiety. A p-value <0.05 was considered statistically significant. ^32^

### Ethical considerations

The Declaration of Helsinki was applied about respect for the confidentiality and accuracy of the data collected during the study, which are presented faithfully. Authorship contributions and transparency in conflicts of interest were reported. ^32, 33^ The project was reviewed and approved by the Ethics Committees of both La Libertad Healthcare Network and the School of Medicine – UCV. Certificate No. 29 issued by the Research Ethics Committee of La Libertad Healthcare Network on February 10, 2025, and Favorable Opinion of the Research Ethics Committee of Medicine – UCV on June 5, 2025. Approval was obtained from the ethics committees prior to initiating this study.

## III. Results

The Table 1 shows that females predominate in both groups (60% in peritoneal dialysis versus 52.2% in hemodialysis). Patients on peritoneal dialysis were married (60%), employed (50%), and had a higher level of education (57.5%). Patients on hemodialysis were single (47.8%), unemployed (44.8%), and had a primary or secondary level of education (37.3% for each level).

**Table 1.** Demographic and clinical profiles according to type of dialysis in patients with chronic kidney disease.

		Group			
		Haemodyalisis		Peritoneal dialysis	
		N°	%	N°	%
Sex	Male	32	47.8%	16	40.0%
	Female	35	52.2%	24	60.0%
Marital status	Single	32	47.8%	11	27.5%
	Married	23	34.3%	24	60.0%
	Divorced-Widowed	12	17.9%	5	12.5%
Employment status	Working	17	25.4%	20	50.0%
	Retired	18	26.9%	14	35.0%
	Unemployed	30	44.8%	2	5.0%
	Housewife	2	3.0%	4	10.0%
Educational level	Illiterate	3	4.5%	0	0.0%
	Primary	25	37.3%	4	10.0%
	Secondary	25	37.3%	8	20.0%
	Technical	3	4.5%	5	12.5%
	Higher	11	16.4%	23	57.5%
Primary cause of CKD	Diabetes Mellitus	21	31.3%	11	27.5%
	Hypertension	31	46.3%	17	42.5%
	Nephritis	2	3.0%	0	0.0%
	Glomerulonephritis	3	4.5%	2	5.0%
	Unknown	10	14.9%	5	12.5%
	Other	0	0.0%	5	12.5%
Comorbidities	Diabetes mellitus	4	6.0%	2	5.0%
	Heart disease/hyperten-sion	13	19.4%	19	47.5%
	Cancer	0	0.0%	0	0.0%
	Peripheral vascular disease	8	11.9%	3	7.5%
	Cerebral vascular disease	1	1.5%	1	2.5%
	Hepatitis C	0	0.0%	0	0.0%
	None	38	56.7%	8	20.0%
	Other	3	4.5%	7	17.5%
Sleep disorders	Yes	31	46.3%	28	70.0%
	No	36	53.7%	12	30.0%
N° of medicines	1–3	34	50.7%	9	22.5%
	4–6	31	46.3%	24	60.0%
	> 6	2	3.0%	7	17.5%
Anxiety according to HEADS score		7.885 ± 4.68		8.725 ± 4.72	
Depression according to HEADS score		9.833 ± 3.43		8.325 ± 4.21	

The main primary causes of CKD in both groups were hypertension and type 2 diabetes mellitus. In terms of comorbidities, more than half of the patients on hemodialysis had no associated diseases (56.7%), while in peritoneal dialysis, heart disease and hypertension were the most prevalent (47.5%).

Sleep disorders were more common in patients undergoing peritoneal dialysis (70%) than in those undergoing hemodialysis (46.3%), as was a higher drug burden (4 to 6 drugs), whereas in hemodialysis the use of 1 to 3 drugs was more common. Moderate to severe anxiety symptoms were more common in the peritoneal dialysis group (37.5%) than in the hemodialysis group (29.9%), while hemodialysis patients showed a higher proportion of moderate to severe depressive symptoms (41.8%).

In Table 2, 27.1% of men and 37.3% of women presented moderate to severe symptoms of anxiety. Most single people (41.9%) showed moderate to severe symptoms of anxiety, as well as 27.7% of married people and 23.5% of divorced or widowed people. According to educational level, one-third of patients at each level – except for those with technical qualifications – presented with moderate to severe anxiety symptoms. 50% of people whose occupation was ‘housewife’ also experienced moderate to severe anxiety symptoms; however no significant association was found between anxiety and sociodemographic characteristics (sig > 0.05).

**Table 2.** Depression and anxiety profiles and their association with demographic variables: gender, age, marital status, employment status, and educational level.

		Anxiety						
		No symptoms		Minor symptoms		Moderate - severe		
		N°	%	N°	%	N°	%	
Sex	Male	23	47.9%	12	25.0%	13	27.1%	Chi:1.258 (p:0.533)
	Female	24	40.7%	13	22.0%	22	37.3%	
Age	<60 years	24	40.7%	16	27.1%	19	32.2%	Chi:1.119 (p:0.571)
	> = 60 years	23	47.9%	9	18.8%	16	33.3%	
Marital status	Single	15	34.9%	10	23.3%	18	41.9%	Chi:3.643 (p:0.457)
	Married	24	51.1%	10	21.3%	13	27.7%	
	Divorced-Widowed	8	47.1%	5	29.4%	4	23.5%	
Employment status	Working	14	37.8%	10	27.0%	13	35.1%	Chi:3.963 (p:0.682)
	Retired	17	53.1%	7	21.9%	8	25.0%	
	Unemployed	13	40.6%	8	25.0%	11	34.4%	
	Housewife	3	50.0%	0	0.0%	3	50.0%	
Educational level	Illiterate	0	0.0%	2	66.7%	1	33.3%	Chi:6.822 (p:0.556)
	Primary	14	48.3%	6	20.7%	9	31.0%	
	Secondary	16	48.5%	6	18.2%	11	33.3%	
	Technical	4	50.0%	3	37.5%	1	12.5%	
	Higher	13	38.2%	8	23.5%	13	38.2%	

		Depression						
		No symptoms		Minor symptoms		Moderate - severe		
		N°	%	N°	%	N°	%	
Sex	Male	10	20.8%	14	29.2%	24	50.0%	Chi:4.914 (p:0.086)
	Female	23	39.0%	17	28.8%	19	32.2%	
Age	<60 years	19	32.2%	20	33.9%	20	33.9%	Chi:2.475 (p:0.290)
	> = 60 years	14	29.2%	11	22.9%	23	47.9%	
Marital status	Single	8	18.6%	16	37.2%	19	44.2%	Chi:9.050 (p:0.060)
	Married	20	42.6%	8	17.0%	19	40.4%	
	Divorced -Widowed	5	29.4%	7	41.2%	5	29.4%	
Employment status	Working	9	24.3%	18	48.6%	10	27.0%	Chi:15.999 (p:0.014)
	Retired	9	28.1%	9	28.1%	14	43.8%	
	Unemployed	11	34.4%	4	12.5%	17	53.1%	
	Housewife	4	66.7%	0	0.0%	2	33.3%	
Educational level	Illiterate	0	0.0%	2	66.7%	1	33.3%	Chi:11.714 (p:0.164)
	Primary	6	20.7%	11	37.9%	12	41.4%	
	Secondary	11	33.3%	7	21.2%	15	45.5%	
	Technical	5	62.5%	3	29.2%	0	0.0%	
	Higher	11	32.4%	8	28.8%	15	44.1%	

In the case of depression, 50% of men and 32.2% of women showed moderate to severe symptoms. Patients aged over 60 whose marital status was ‘single’ or ‘married’ mostly presented with moderate to severe symptoms. According to educational level, 66.7% of illiterate patients showed mild symptoms, while moderate and severe symptoms predominated in patients with primary and secondary education. In terms of employment status, 48.6% of active workers showed mild symptoms, while moderate and severe symptoms predominated in retirees and the unemployed (sig < 0.05).

In Table 3, thirty-nine percent of patients with sleep disorders had moderate to severe anxiety symptoms, and among those without sleep disorders, 25% also experienced symptoms of that severity. Regarding the number of medications, 55.6% of those who consume more than six medications show severe symptoms of anxiety. However, no statistically significant association was found (p > 0.05).

**Table 3.** Depression and anxiety and their association with sleep disorder variables and medication quantity.

		Anxiety						
		No symptoms		Minor symptoms		Moderate - severe		
		N°	%	N°	%	N°	%	
Sleep disorders	Yes	24	40.7%	12	20.3%	23	39.0%	Chi:2.413 (p:0.299)
	No	23	47.9%	13	27.1%	12	25.0%	
N° of medicines	1–3	20	46.5%	8	18.6%	15	34.9%	Chi:4.059 (p:0.398)
	4–6	24	43.6%	16	29.1%	15	27.3%	
	> 6	3	33.3%	1	11.1%	5	55.6%	

		Depression						
		No symptoms		Minor symptoms		Moderate - severe		
		N°	%	N°	%	N°	%	
Sleep disorders	Yes	15	25.4%	17	28.8%	27	45.8%	Chi:2.270 (p:0.321)
	No	18	37.5%	14	29.2%	16	33.3%	
N° of medicines	1–3	10	23.3%	13	30.2%	20	46.5%	Chi:3.577 (p:0.466)
	4–6	19	34.5%	17	30.9%	19	34.5%	
	> 6	4	44.4%	1	11.1%	4	44.4%	

Regarding depression and its relationship with the variables described above, it was found that 45.8% of those with sleep disorders showed moderate to severe symptoms of depression and 28.8% showed mild symptoms. Regarding the amount of medication, 46.5% of those who took 1 to 3 medications or more than 6 medications showed moderate to severe symptoms of depression; with no evidence of association (p > 0.05).

In Table 4, about the variables age and duration of dialysis, there were no differences among the study groups. However, significant differences were observed in several biochemical parameters, such as albumin (t: 2.767; p: 0.003) and hemoglobin values, which are higher in hemodialysis patients (t: 2.326; p: = .012), with a moderate effect size (d = 0.504). With regard to urea, the highest levels are found in peritoneal dialysis patients (t:-1.956; p: .027), as are calcium values (t: −1.669; p: .049) and parathyroid hormone levels (t: −2.391; p: .011), with mainly moderate effect sizes. These findings suggest that biochemical profiles differ according to dialysis modality.

**Table 4.** Comparison of laboratory variables according to type of dialysis in patients with end-stage renal disease.

	Type of dialysis	N	Media	Standard deviation	Test t
Age	Haemodialysis	67	55.134	15.43	−1.027 (p: .153)
	Peritoneal dialysis	40	58.325	15.75	d de Cohen (−.205)
Duration of dialysis (years)	Haemodialysis	66	5.106	5.24	−.759 (p: .225)
	Peritoneal dialysis	40	5.950	6.02	d de Cohen(−.152)
Albumin 3,8–5,0 g/dL	Haemodialysis	67	3.951	.48	**2.767 (p: .003)**
	Peritoneal dialysis	40	3.685	.46	**d de Cohen(.553)**
Urea 12–54 mg/dL	Haemodialysis	67	128.247	45.77	**−1.956 (p: .027)**
	Peritoneal dialysis	40	165.050	142.54	**d de Cohen(−.391)**
Hemoglobin >12 g/dL (Women) >13 g/dL (Men)	Haemodialysis	67	10.494	1.11	**2.326 (p: .012)**
	Peritoneal dialysis	40	9.842	1.55	**d de Cohen(.504)**
Serum calcium 8,5–10,5 mg/dL	Haemodialysis	67	8.441	1.67	**−1.669 (p: .049)**
	Peritoneal dialysis	40	9.036	1.97	**d de Cohen(−.334)**
Phosphorus 2,5–4,5 mg/dL	Haemodialysis	67	4.989	1.69	−1.594 (p: .057)
	Peritoneal dialysis	40	5.544	1.83	d de Cohen(−.318)
Parathyroid hormone 10–55 pg/mL	Haemodialysis	67	201.292	149.96	**−2.391 (p: .011)**
	Peritoneal dialysis	40	514.895	821.50	**d de Cohen(−.609)**

Table 5: In relation to anxiety, only hemoglobin showed a statistically inverse correlation (rho = −0.197; p = 0.042), indicating that lower hemoglobin levels were associated with higher anxiety levels. No significant relationship was observed among anxiety and dialysis duration, albumin, urea, calcium, phosphorus, or parathyroid hormone (p > 0.05).

**Table 5.** Laboratory variables in dialysis patients and their association with depression and anxiety.

			Anxiety	Depression
Spearman’s rho	Duration of dialysis (years)	Correlation coefficient	−0.061	−0.048
		Sig. (bilateral)	0.533	0.627
		N	106	107
	Albumin 3,8–5,0 g/dL	Correlation coefficient	−0.080	−0.053
		Sig. (bilateral)	0.413	0.590
		N	107	107
	Urea 12–54 mg/dL	Correlation coefficient	−0.082	**−.204** ^*^
		Sig. (bilateral)	0.402	0.036
		N	107	107
	Hemoglobin >12 g/dL (Women) >13 g/dL (Men)	Correlation coefficient	**−.197** ^*^	**−.199** ^*^
		Sig. (bilateral)	0.042	0.041
		N	107	107
	Calcium 8,5–10,5 mg/dL	Correlation coefficient	−0.137	−0.172
		Sig. (bilateral)	0.159	0.078
		N	107	107
	Phosphorus 2,5–4,5 mg/dL	Correlation coefficient	−0.053	**−.228** ^*^
		Sig. (bilateral)	0.590	0.019
		N	107	107
	Parathyroid hormone 10–55 pg/mL	Correlation coefficient	0.028	−0.039
		Sig. (bilateral)	0.776	0.691
		N	107	107

About depression, a relationship was identified with urea (rho = −0.204; p = 0.036), hemoglobin (rho = −0.199; p = 0.041), and phosphorus (rho = −0.228; p = 0.019), indicating that when hemoglobin and phosphorus levels decrease, depression levels increase. The duration of dialysis, albumin, calcium, and parathyroid hormone did not show association with depression (p > 0.05).

Table 6: The ordinal logistic regression model showed an acceptable fit (Nagelkerke’s R ^2^ = 0.274). After adjustment, older age remained independently associated with greater anxiety severity (β = 0.047; 95% CI: 0.006–0.088; p = 0.023), whereas higher serum calcium levels were independently associated with lower anxiety severity (β = −0.616; 95% CI: −1.050 to −0.182; p = 0.005).

**Table 6.** Multivariate ordinal regression analysis to estimate factors associated with anxiety.

		Estimate	Std. Error	Wald	Df	Sig.	95% Confidence interval	
							Lower bound	Upper bound
Threshold	[Anxiety = 0]	−3,389	2,608	1,689	1	,194	−8,501	1,722
	[Anxiety = Mild]	−2,246	2,599	,747	1	,387	−7,339	2,847
Location	Age	,047	,021	5,143	1	**,023**	,006	,088
	Duration of dialysis	,018	,038	,237	1	,627	-,056	,093
	Calcium	-,616	,222	7,736	1	**,005**	−1,050	-,182
	Urea	-,001	,004	,032	1	,858	-,009	,008
	[Marital status = Single]	1,302	,674	3,736	1	,053	-,018	2,622
	[Marital status = Married]	,658	,630	1,092	1	,296	-,576	1,893
	[Marital status = divorced/ widowed]	0 ^a^	.	.	0	.	.	.
	[Employment status = Active]	-,634	,913	,482	1	,488	−2,424	1,156
	[Employment status = Retired]	−1,901	,971	3,831	1	,050	−3,805	,003
	[Employment status = Unemployed]	-,896	,958	,874	1	,350	−2,774	,982
	[Employment status = Housewife]	0 ^a^	.	.	0	.	.	.
	[Comorb = T2D]	−1,703	1,091	2,435	1	,119	−3,842	,436
	[Comorb = Cardiac disease]	-,358	,786	,207	1	,649	−1,899	1,184
	[Comorb = Cancer]	-,873	,940	,864	1	,353	−2,715	,968
	[Comorb = Vascular disease]	20,463	,000	.	1	.	20,463	20,463
	[Comorb = Hepat C]	-,681	,752	,820	1	,365	−2,155	,793
	[Comorb = 0]	0 ^a^	.	.	0	.	.	.
	[Sleep disorder = Yes]	,635	,401	2,502	1	,114	-,152	1,422
	[Sleep disorder = No]	0 ^a^	.	.	0	.	.	.

Liaison function: Logit

Nagelkerke’s R: .274.

^a^ This parameter is set to zero because it is redundant.

Table 7: The multivariable ordinal logistic regression model showed an acceptable overall fit (Nagelkerke’s R ^2^ = 0.279), explaining 27.9% of the variability in depressive symptom severity. After adjustment, older age (β = 0.051; 95% CI: 0.010–0.091; p = 0.014), single marital status compared with being divorced (β = 1.380; 95% CI: 0.090–2.669; p = 0.036), sleep disorders (β = 0.985; 95% CI: 0.192–1.778; p = 0.015), cancer (β = 1.970; 95% CI: 0.092–3.848; p = 0.040), and hepatitis C (β = 1.570; 95% CI: 0.126–3.015; p = 0.033) were independently associated with greater depressive symptom severity, whereas higher serum calcium levels were associated with lower depressive symptom severity (β = −0.359; 95% CI: −0.645 to −0.073; p = 0.014).

**Table 7.** Multivariate ordinal regression analysis to estimate factors associated with depression.

		Estimate	Std. Error	Wald	df	Sig.	95% Confidence interval	
							Lower bound	Upper bound
Threshold	[Depression = 0]	2,962	2,086	2,016	1	,156	−1,127	7,050
	[Depression = Mild]	4,417	2,113	4,370	1	,037	,276	8,559
Location	Age	,051	,021	6,085	1	**,014**	,010	,091
	Duration of dialysis	-,024	,037	,410	1	,522	-,096	,049
	Calcium	-,359	,146	6,060	1	**,014**	-,645	-,073
	Urea	,003	,003	1,029	1	,310	-,003	,009
	[Marital status = Single]	1,380	,658	4,396	1	**,036**	,090	2,669
	[Marital status = Married]	,536	,587	,833	1	,361	-,615	1,687
	[Marital status = divorced/ widowed]	0 ^a^	.	.	0	.	.	.
	[Employment status = Active]	1,076	,957	1,266	1	,261	-,799	2,952
	[Employment status = Retired]	,851	,998	,726	1	,394	−1,106	2,808
	[Employment status = Unemployed]	1,618	,994	2,648	1	,104	-,331	3,566
	[Employment status = Housewife]	0 ^a^	.	.	0	.	.	.
	[Comorb = T2D]	1,293	,994	1,692	1	,193	-,655	3,241
	[Comorb = Cardiac disease]	1,418	,777	3,328	1	,068	-,105	2,942
	[Comorb = Cancer]	1,970	,958	4,225	1	**,040**	,092	3,848
	[Comorb = Vascular disease]	1,094	1,813	,364	1	,546	−2,460	4,648
	[Comorb = Hepat C]	1,570	,737	4,539	1	**,033**	,126	3,015
	[Comorb = 0]	0 ^a^	.	.	0	.	.	.
	[Sleep disorder = Yes]	,985	,404	5,930	1	**,015**	,192	1,778
	[Sleep disorder = No]	0 ^a^	.	.	0	.	.	.

Liaison function: Logit

Nagelkerke’s R: .279.

^a^ This parameter is set to zero because it is redundant.

## IV. Discussion

A total of 107 patients with stage G5 chronic kidney disease (CKD) undergoing dialysis during the study period were studied; 67 were on hemodialysis (48% men and 52% women) and 40 were on peritoneal dialysis (40% men and 60% women). The average age of the patients was 55.134 ± 15.43 and 58.325 ± 15.75 years, respectively.

CKD is a major health problem that negatively affects patients’ quality of life and is associated with various psychiatric conditions. ^12^ Patients with CKD are believed to experience chronic stress that increases as kidney function deteriorates. ^3^ In addition, dietary restrictions, comorbidities, adverse effects of medications, changes in self-perception, and fear of death become more pronounced during the advanced stages of the disease. ^13^ Consequently, patients undergoing hemodialysis are at greater risk of developing mental health disorders, mood disturbances, functional impairment, and other psychological complications. ^12^^–^^15^

Mental health problems including anxiety, ^1^^–^^3^^,^^6^^,^^11^^,^^15^^,^^20^ depression ^1^^,^^2^^,^^9^^–^^11^^,^^20^ and sleep disorders ^3^^,^^5^^,^^14^ have been widely reported among patients with stage G5 CKD. Previous studies have described these conditions in relation to demographic characteristics, such as age, marital status, employment status, educational level, as well as clinical variables. One study reported that patients receiving hemodialysis were predominantly male with a mean age of 59.1 ± 16 years, and were more likely to be married, illiterate, and unemployed. ^1^ Another study found that 72.31% of patients with CKD were married or living with a partner. ^34^ In the present study, women predominated in both dialysis groups, with a higher proportion among patients in peritoneal dialysis. Patients undergoing hemodialysis were more frequently single, unemployed, and had primary and secondary education, whereas those receiving peritoneal dialysis were commonly married, employed, and had higher educational attainment.

Regarding the primary causes of end-stage renal disease (ESRD), studies have identified diabetes, ^1^^,^^35^ glomerulonephritis, ^35^ hypertension, ^1^^,^^35^ and vascular nephropathy as the most frequent etiologies. ^35^ However, the underlying cause remains unknown in approximately 25.6% of cases. ^1^ Diabetes mellitus and hypertension share common pathophysiological mechanisms involved in the development and progression of ESRD ^36^^,^^37^ and the microvascular complications associated with DM are largely responsible for progression to stage G5 CKD requiring dialysis or kidney transplantation. ^38^^,^^39^ Likewise, high blood pressure has been recognized as both a cause and a consequence of CKD. ^40^ In the present study, hypertension and type 2 diabetes mellitus were the leading primary causes of end-stage CKD, regardless of dialysis modality. The primary cause of CKD remained unknown in 15% of patients undergoing hemodialysis and in 12.5% of those receiving peritoneal dialysis; proportions that are lower than those previously reported, where the primary cause of CKD remained unidentified in up to 25.6% of patients. ^1^

Diabetes mellitus, hypertension and a history of cardiovascular disease have been reported among the most common comorbidities in patients with CKD. ^35^^,^^41^ In the present study, heart disease/hypertension and sleep disorders were more frequent among patients undergoing peritoneal dialysis, whereas more than 56% of patients receiving hemodialysis had no comorbidities; among those with comorbid conditions, heart disease and peripheral vascular disease were the most common. Regarding sleep disorders, one study have shown that insomnia is associated not only with comorbidities but also with advanced age, fatigue, and pruritus. ^42^ Patients undergoing, peritoneal dialysis also had a higher medication burden, with most receiving four to six medications, whereas those undergoing hemodialysis more frequently used one to three medications.

It has been reported that one in five patients with CKD experience depression or anxiety at the initiation of dialysis. ^1^ In a cross-sectional study in Morocco, the prevalence of anxiety among patients undergoing hemodialysis ranged from 25.2% to 69.3%, while the prevalence of depression ranged from 34% to 67%. ^35^ Another Moroccan study reported anxiety and depression prevalences of 28.2% and 23.1%, respectively, among patients receiving the same treatment. ^9^ In Peru, previous studies have identified depression in patients undergoing hemodialysis, with mild depression being the most common presentation (48.9%). ^43^ In the present study, the prevalence of anxiety symptoms was 56% whereas depressive symptom were observed in 69.15% of patients, regardless of dialysis modality. In all these studies, the HADS ^21^^–^^23^ questionnaire was used to detect anxiety and depression symptoms and assess their severity in dialysis patients. ^22^^,^^23^ Compared with other screening instruments the HEADS i considered more specific because it minimizes the influence of physical symptoms that may overlap with psychological manifestations. ^20^

In a previous study using the HADS, the mean anxiety score among patients undergoing hemodialysis was 7.7 ± 4.6 while the mean depression score was 7.4 ± 4.2. ^1^ In the present study, patients receiving hemodialysis had a mean anxiety score of 7.985 ± 4.675 and a mean depression score of 9.833 ± 3.431, indicating similar anxiety levels but higher depressive symptoms scores. Among patients on peritoneal dialysis, the mean anxiety and and depression scores were 8.725 ± 4.723 and 8.325 ± 4.208, respectively. These findings differ from those reported in a study conducted in India using the same instrument, in which patients undergoing hemodialysis had mean anxiety and depression scores of 13.82 ± 2.73 and 12.58 ± 3.12, respectively. The authors suggested that these differences may be related to socioeconomic and healthcare disparities, including limited access to health services, and lower educational attainment, factors that have been associated with psychological distress among patients undergoing dialysis. ^3^ Despite these differences, both studies reported a high burden of psychological symptoms among patients undergoing dialysis.

When stratified by dialysis modality, depressive symptoms were more frequent than anxiety symptoms among patients undergoing hemodialysis, with 41.8% presenting moderate to severe depression symptoms. In contrast, moderate to severe anxiety symptoms were more common among patients receiving peritoneal dialysis. Similar findings have been reported in previous studies, which indicate that up to 74% of patients undergoing hemodialysis experience depressive symptoms ^9^ and that depression is more prevalent in individuals receiving chronic hemodialysis than in those undergoing peritoneal dialysis (30.6% vs. 20.4%, p = 0.04). ^8^

Another study reported anxiety symptoms in 41.79% of patients with advanced CKD, of whom 13.43% had a clinical diagnosis of anxiety. ^34^ However, that study included patients with CKD stage G3 and above, whereas the present study was limited to patients with stage G5 CKD undergoing dialysis, a population expected to have a greater psychological burden. The same study reported depressive symptoms in 25.38% of patients, ^34^ compared with 41.8% of moderate to severe depressive symptoms observed among patients undergoing hemodialysis in the present study.

Psychological symptoms have also been described among patients receiving peritoneal dialysis, particularly in those who previously underwent hemodialysis before switching because of vascular access exhaustion. ^45^ This previous experience may influence patients’ perceptions of their disease and treatment. ^45^ In addition, the home-based nature of peritoneal dialysis may contribute to concerns about managing potential complications without immediate medical assistance, whereas the hospital setting of hemodialysis may provide a greater sense of security. Nevertheless, these explanations remain speculative, and further research is needed to better understand the psychological factors associated with different dialysis modalities.

When examining the association between demographic variables, including sex, age, marital status, employment status, and educational level and anxiety symptoms, more than 50% of both women and men reported some degree of anxiety. Regarding the severity of symptoms, 37.3% of women and single people presented moderate to severe symptoms of anxiety, while mild anxiety symptoms predominated among individuals with no formal education. However, no statistically significant association were found between these variables and anxiety symptoms. This finding differs from previous studies that reported significant associations between age and sleep disorders and anxiety, ^1^ between lower educational level and higher levels of depression and anxiety. ^43^

Likewise, 61% of women and 79% of men experienced some degree of depressive symptoms. Moderate to severe depressive symptoms were observed in 50% of men, one-third of women, individuals older than 60 years of age, single participants, those with primary or secondary education, retirees, and unemployed individuals. Among married people, 40.4% reported moderate to severe symptoms, whereas mild symptoms predominated among individuals with no formal education and those who were actively employed. Similarly, a Peruvian study reported a higher prevalence of moderate depression among older adults, particularly widowed individuals, followed by divorced and single patients. These findings were attributed to the absence of a partner and limited social support for coping with the disease and attending dialysis sessions. ^43^

In the present study employment status showed a weak but statistically significant association with depressive symptoms. Other studies have likewise reported associations between unemployment and depression, ^45^^,^^46^ as well as between depression and advanced age, ^1^^,^^43^ lower educational level, ^1^^,^^10^^,^^43^^,^^47^ retirement and poor financial status. ^45^ Unemployment has been associated with depression because it involves stressors related to economic autonomy and makes patients more vulnerable to disease; ^43^ however employment status is only one of several factors influencing depression in patients receiving dialysis.

Other studies have also reported associations between depression and marital status, educational level, ^1^^,^^43^ and socioeconomic status, ^1^ findings that differ from those observed in the present study. In addition, patients with lower educational attainment have been reported to be more likely to experience depressive symptoms than those with higher educational levels. ^43^

A systematic review of 28 studies confirmed that depression and anxiety are highly prevalent among patients with CKD receiving dialysis ^44^; consistent with the high frequency of both conditions observed in our study. Because anxiety and depression are closely linked to CKD particularly in patients requiring dialysis, ^3^ psychosocial factors such as family support and coping strategies should also be considered when assessing psychological well-being. ^44^

Sleep disorders are among the factors that negatively affect the quality of life of patients with CKD, with a reported prevalence ranging from 41% to 83% depending on the assessment instrument used. They commonly manifest as difficulty initiating or maintaining sleep, excessive daytime sleepiness, and chronic fatigue. ^3^ In the present study, moderate to severe anxiety and depressive symptoms were observed in 39% and 45.8% of patients with sleep disorders, respectively. Previous studies have likewise identified sleep disorders as factors associated with anxiety in patients with CKD. ^1^

Another study, reported significantly higher levels of insomnia among individuals older than 60 years, divorced or widowed, patients with comorbidity, individuals experiencing fatigue after hemodialysis, or persistent fatigue, and those who had pruritus or joint stiffness. ^42^ An association has also been reported among anxiety, depression, and poor sleep quality in older adults with albuminuria and – reduced glomerular filtration rate, although the underlying mechanism remain unclear, these relationships may be partly explained by hormonal alterations associated with CKD. ^48^ In addition, sleep disorders have been linked to changes in corticothalamic activity and increased concentrations of adrenocorticotropic hormone and cortisol, which may impair both sleep initiation and sleep maintenance. Consequently, poor sleep quality has been associated with higher levels of anxiety and depression. ^49^^,^^50^

Among patients with moderate to severe anxiety symptoms, polypharmacy (defined as the use of more than six medications) was common. However, no statistically significant association was found between this variable and symptoms of anxiety and depression. This finding differs from that of another study in which anxiety and depression scores were significantly correlated with the number of prescribed medications (P = 0.022 and P = 0.003, respectively). ^1^ Polypharmacy is particularly common among older adults with CKD and has been associated with an increased risk of adverse drug reactions, cumulative toxicity, drug interactions and functional decline. ^51^ Although polypharmacy was not independently associated with anxiety or depression in our study, its high prevalence among patients with CKD underscores the importance of regular medication review to minimize treatment burden and reduce the risk of adverse clinical outcomes.

It has been reported that dialysis-related factors, including treatment modality, travel time, waiting time treatment setting, dietary restrictions, limited family or social support, and unemployment may contribute to emotional distress and increase the risk of depression and anxiety. In addition, older age and longer time on dialysis have been associated with higher levels of depression. ^43^ In contrast, the present study, found no significant differences in age or dialysis duration and symptoms of anxiety or depression. So, in our study population, demographic characteristics and dialysis duration were not major determinants of psychological symptoms, highlighting the potential influence of other clinical and psychosocial factors.

Regarding the biochemical parameters evaluated, patients undergoing hemodialysis showed significantly higher albumin (t: 2.767; p: 0.003) and hemoglobin levels (t: 2.326; p = .012), with a moderate effect size (Cohen’s d = 0.504). In contrast, patients receiving peritoneal dialysis had significantly higher urea (t: −1.956; p: .027); calcium (t: −1.669; p: .049) and parathyroid hormone levels (t: −2.391; p: .011), with predominantly moderate effect size. These findings suggest that dialysis modality may influence the biochemical profile of patients. Previous studies have reported a high prevalence of hyperphosphatemia among patients receiving dialysis, ^52^ which may be related to poor adherence to phosphate binder therapy and the complexity of dietary phosphate restriction, factors that have also been associated with depressive symptoms. ^44^ In the present study, mean phosphorus levels were 4.989 mg/dL in patients undergoing hemodialysis and 5.544 mg/dL in those receiving peritoneal dialysis. These findings are comparable to those of another study, in which 68.2% of patients with end-stage CKD had phosphorus levels ≥5.5 mg/dL, ^44^ however, their direct contribution to psychological symptoms remains uncertain and should be interpreted alongside other clinical and psychosocial factors.

Regarding anxiety, hemoglobin was the only biochemical marker that showed a statistically significant, although weak, inverse association with anxiety symptoms. This finding is consistent with previous reports suggesting that anemia-related alterations may contribute to psychological distress in patients with CKD. ^53^ However, the small effect size indicates that hemoglobin alone is unlikely to explain anxiety symptoms, which are probably influenced by multiple biological and psychosocial factors. Low hemoglobin levels caused by insufficient erythropoietin production are common in CKD, and their symptoms could be confused with those of depression. ^54^

No significant associations were found between anxiety symptoms and dialysis duration, serum albumin, urea, calcium, phosphorus, or parathyroid hormone. These findings are consistent with previous studies reporting no significant associations between dialysis duration, serum calcium, phosphorus levels and symptoms of anxiety and depression. ^52^Another study reported that patients with generalized anxiety disorder and elevated parathyroid hormone levels have poor sleep quality and higher levels of anxiety, suggesting that disturbances in calcium homeostasis, including elevated parathyroid hormone levels, may be associated with poor sleep quality and anxiety symptoms. ^55^ However, this association was not observed in our study.

In the present study, depressive symptoms showed weak inverse correlations with serum urea (rho = −0.204; p = 0.036), hemoglobin (rho = −0.199; p = 0.041), and serum phosphorus levels (rho = −0.228; p = 0.019). Similarly, previous studies have reported significant associations between depressive symptoms and glomerular filtration rate (GFR), serum urea, and hemoglobin, as well as between anxiety symptoms and diabetes mellitus and serum urea. ^35^ Another study also found that anxiety and depression scores were negatively correlated with GFR, hemoglobin, and serum calcium; and positively correlated with white blood cell count, serum urea, creatinine, and phosphate levels. ^3^ Although these associations were statistically significant, their magnitude was generally weak, suggesting that biochemical parameters alone are unlikely to explain the occurrence of depressive symptoms in patients with CKD undergoing dialysis.

A longitudinal study involving more than 2,000 patients with CKD undergoing hemodialysis and diagnosed with depression, reported that serum urea levels were more strongly associated with depressive symptoms than with depressive disorders, although this association did not reach statistical significance. ^56^ Elevated serum urea levels in end-stage CKD have been associated with accelerated atherosclerosis which may contribute to brain aging and associated pathologies such as depression and neurocognitive disorders. ^55^^,^^56^ In addition, persistent elevation of urea in CKD promotes protein carbamylation, which has been associated with vascular and inflammatory damage. ^55^ These mechanisms, together with the neurotoxic effects of uremic toxins and chronic systemic inflammation, have been proposed as potential contributors to depression and other neurological complications in patients with CKD. ^7^^,^^58^

The association between CKD and the high prevalence of cerebrovascular disease, cognitive impairment, and depression has been well documented. ^7^^,^^56^ Uremic toxins have also been implicated in the development of some neurological disorders, as they have direct neurotoxic actions such as astrocyte activation and neuronal death, and indirect actions via vascular effects such as endothelial dysfunction, calcification, ^7^ and chronic inflammation. ^7^^,^^57^^,^^58^ Among these toxins, indoxyl sulfate, has demonstrated well-recognized neurotoxic effects. ^7^ On the other hand, CKD is a complex pathology characterized by chronic inflammation, oxidative stress, and vascular injury, which, together with dysregulation of the hypothalamic-pituitary-adrenal axis, are thought to contribute to pathogenesis of depression. ^58^

Regarding hemoglobin and phosphorus levels, this study found inverse associations between these biochemical parameters and the severity of depressive symptoms. As previously discussed, anemia is a common complication in patients with CKD undergoing dialysis and may result from reduced erythropoietin production, erythropoietic depressants induced by uremic toxins, iron deficiency, and the shortened lifespan of erythrocytes. Anemia is significantly associated with depression in patients with CKD particularly among older patients and have also suggested that low albumin levels may contribute to this association. ^50^ Although the associations observed in our study were statistically significant, their magnitude was weak, indicating that hemoglobin and phosphorus levels alone are unlikely to fully explain depressive symptoms, which are probably influenced by multiple biological and psychosocial factors.

Previous studies have reported a significant association between nutritional status, as assessed by serum albumin levels, and depressive symptoms in patients with CKD. ^59^ In contrast the present study found no significant association between serum albumin levels and symptoms of anxiety or depression. Regarding phosphorus, elevated serum levels have been associated with poor adherence to phosphate binders and the complexity of dietary phosphate restriction, both of which have been linked to depressive symptoms. ^44^ Although phosphorus was inversely associated with depressive symptoms in our study, the strength of this association was weak, suggesting that phosphorus levels alone are unlikely to account for the psychological burden experienced by patients with CKD.

The regression analysis identified older age and lower serum calcium levels as independent correlates of greater anxiety severity in patients with CKD undergoing dialysis. Older age has consistently been associated with a higher prevalence of anxiety symptoms among patients with CKD, possibly reflecting the cumulative burden of comorbidities, functional decline, and psychosocial stress associated with aging. ^1^^,^^43^ About calcium levels, our finding is consistent with a previous study reporting an inverse correlation between serum calcium levels and both anxiety and depression scores in patients with CKD. ^3^ Although these associations were statistically significant, their magnitude was modest, suggesting that age and calcium are only part of the complex biological and psychosocial factors contributing to anxiety in patients undergoing dialysis. Therefore, these findings should be interpreted cautiously and confirmed in larger prospective studies.

The multivariable analysis identified older age, single marital status, sleep disorders, cancer, hepatitis C, and lower serum calcium levels as independent correlates of greater depressive symptom severity. However, the observed associations were generally modest, suggesting that depressive symptoms in patients with CKD are likely influenced by multiple biological, clinical, and psychosocial factors.

Previous studies reported a weak inverse association between age and depression and have found that women tend to report higher levels of anxiety and depression than men. ^60^ The females had higher levels of depression, anxiety, and stress and experienced decreases in albumin, calcium, and vitamin D levels, suggesting that sex may influence the relationship between inflammation, nutritional status, and psychological distress in patients undergoing hemodialysis. ^61^ The magnitude of the association was modest, suggesting that demographic and biochemical factors alone are insufficient to explain the psychological burden experienced by patients with CKD.

Depression may also lead to behavioral changes that adversely affect medical outcomes. Among patients with CKD undergoing dialysis, depressive symptoms has been associated with poor adherence to pharmacological treatment, non-adherence to dietary recommendations, weight gain between dialysis sessions, and non-attendance at dialysis sessions. ^57^ These findings underscore the importance of adopting a comprehensive, patient-centered approach to CKD management that extends beyond the traditional biomedical model. In addition to optimizing medical treatment, strategies aimed at reducing anxiety and depression—such as lifestyle modification, cognitive behavioral therapy, regular physical activity, relaxation techniques, and mindfulness-based interventions—may improve treatment adherence and overall clinical outcomes. ^1^^,^^44^^,^^62^

Current research reports several factors associated with depression and supports the bidirectional relationship between CKD and depression. ^57^ However, further research is needed to clarify the biological mechanisms underlying depression in patients with CKD, particularly the role of biochemical markers and their potential clinical relevance. In addition, implementing standardized screening protocols at least twice a year, using validated self-administered questionnaires followed by psychological or psychiatric evaluation when indicated may facilitate the early detection and management of anxiety and depression. Healthcare institutions should also strengthen organizational support and provide educational, family, and psychosocial resources to help patients cope with the emotional challenges of dialysis and improve treatment adherence.

### Limitations of the study

This study was subject to the limitations inherent to a cross-sectional design, which precludes establishing causal relationships and is vulnerable to selection bias. Consequently, this study provide only a baseline assessment, highlighting the need for longitudinal follow-up of this cohort, including analysis of polypharmacy, treatment for anxiety or depression, and family and social support. Longitudinal studies are needed to confirm many of the findings observed in the present study. Likewise, although a more parsimonious multivariable regression model was used, the relatively small sample size may have limited the statistical power to detect weaker associations and affected the precision of some estimates.

Additional limitations were encountered in the analysis of biochemical markers. Although these assessments were included in the clinical protocol for patients undergoing dialysis, logistical difficulties at the hospital delayed the collection and evaluation of some laboratory tests.

### Scientific contribution

This study provides original data on the clinical, laboratory and demographic factors associated with anxiety and depression among Peruvian patients with G5 chronic kidney disease undergoing hemodialysis or peritoneal dialysis. Given the limited evidence available in Peru, these findings contribute to a better understanding of the psychosocial challenges faced by this population and may support the development of institutional strategies and updated protocols aimed at improving the comprehensive and interdisciplinary care of patients receiving dialysis.

## V. Conclusions

In this study, symptoms of anxiety and depression were prevalent among patients with G5 CKD undergoing dialysis. Older age and lower serum calcium levels were independently associated with greater anxiety severity, whereas older age, single marital status, sleep disorders, cancer, hepatitis C, and lower serum calcium levels were independently associated with greater depressive symptom severity. Although these associations were statistically significant, their magnitude was generally modest, suggesting that anxiety and depression in patients undergoing dialysis are multifactorial conditions influenced by biological, clinical, and psychosocial factors.

The diagnosis and commencement of dialysis for patients with G5 CKD have a profound emotional impact, forcing patients to drastically alter their lifestyle. This transition often triggers anxiety, depression and chronic stress; psychological states that cause physiological changes — such as elevated cortisol levels and high blood pressure — which accelerate kidney damage. Furthermore, emotional distress compromises adherence to treatment and limits social participation due to a loss of autonomy. Consequently, given that psychological reactions directly influence disease progression and mortality, a multidisciplinary approach is essential. Integrating psychological care from the early stages is key to developing active coping strategies, optimizing mental health and, consequently, safeguarding the patient’s quality of life.

## Data Availability

Zenodo. Prevalence and factors associated with depression and anxiety in patients with chronic kidney disease. Available at https://doi.org/10.5281/zenodo.18968127. ^63^ This project contains the following underlying data:
1. Data collection form 2. Hospital Anxiety and Depression Scale 3. DATABASE ok Anxiety and Depression in ERC.xlsx 4. Informed consent English Data is available under the terms of the Creative Commons Attribution 4.0 International license
