# Supplementary material for: Prevalence and Factors Associated with Depression and Anxiety in Patients with Chronic Kidney Disease
Source: F1000Res. 2026 Aug 8;15:575. Originally published 2026 Apr 19. [Version 3] doi: 10.12688/f1000research.178880.3 (PMC13560567; doi:10.12688/f1000research.178880.3)
Supplement: Peer review history

# Reviewer report: Reviewer response for version 3

**DOI:** 10.5256/f1000research.207390.r509053

**Published:** 2026-08-27

**Authors:** MARIA SOFIA CUBA-FUENTES

**Affiliations:** 1 Universidad Peruana Cayetano Heredia, Lima District, Lima Region, Peru

The authors have satisfactorily addressed the comments and recommendations raised during the review process. The revisions have substantially improved the methodological rigor, clarity, and consistency of the manuscript, particularly with regard to the statistical analyses, interpretation of the findings, and alignment of the language with the cross-sectional study design.

 I have no further major concerns. In my opinion, the manuscript is now suitable for indexing, and I recommend its acceptance.

Is the work clearly and accurately presented and does it cite the current literature?

Partly

If applicable, is the statistical analysis and its interpretation appropriate?

Partly

Are all the source data underlying the results available to ensure full reproducibility?

Yes

Is the study design appropriate and is the work technically sound?

Partly

Are the conclusions drawn adequately supported by the results?

Partly

Are sufficient details of methods and analysis provided to allow replication by others?

Yes

Reviewer Expertise:

Primary Care, health systems

I confirm that I have read this submission and believe that I have an appropriate level of expertise to confirm that it is of an acceptable scientific standard.

# Reviewer report: Reviewer response for version 3

**DOI:** 10.5256/f1000research.207390.r509054

**Published:** 2026-08-24

**Authors:** Hanoch Livneh

**Affiliations:** 1 Portland State University, Portland, Oregon, USA

I completed review of the authors' changes and response to my earlier comments.

 I am satisfied with the thoughtful and detailed revisions they made in the updated version.

 Best,

 Hanoch Livneh (reviewer)

Is the work clearly and accurately presented and does it cite the current literature?

Partly

If applicable, is the statistical analysis and its interpretation appropriate?

Partly

Are all the source data underlying the results available to ensure full reproducibility?

Yes

Is the study design appropriate and is the work technically sound?

Yes

Are the conclusions drawn adequately supported by the results?

Partly

Are sufficient details of methods and analysis provided to allow replication by others?

Yes

Reviewer Expertise:

Psychosocial adaptation to chronic illness and disability

I confirm that I have read this submission and believe that I have an appropriate level of expertise to confirm that it is of an acceptable scientific standard.

# Reviewer report: Reviewer response for version 2

**DOI:** 10.5256/f1000research.204749.r494073

**Published:** 2026-07-15

**Authors:** MARIA SOFIA CUBA-FUENTES

**Affiliations:** 1 Universidad Peruana Cayetano Heredia, Lima District, Lima Region, Peru

This manuscript investigates the prevalence of anxiety and depression and their associated demographic, clinical, and laboratory factors among Peruvian patients with chronic kidney disease undergoing dialysis. The topic is clinically relevant, particularly given the limited evidence available from Latin America. The study contributes valuable local data and addresses an important aspect of comprehensive CKD care.

 The manuscript has improved substantially compared with the previous version. The authors have addressed many of the reviewer’s comments, including clarifying the study design, expanding the limitations section, improving the description of the HADS instrument, and incorporating more cautious language regarding some findings. The manuscript is generally well organized, the methods are sufficiently described, and the underlying data are publicly available, enhancing transparency and reproducibility.

 However, several issues remain that should be addressed before the manuscript is considered fully robust.

**Major comments**

**1. Interpretation of statistical findings**

 Although the authors acknowledge the Bonferroni correction and mention that some associations are no longer statistically significant after adjustment for multiple comparisons, several sections of the Results, Discussion, and Conclusions continue to emphasize nominally significant findings (e.g., p values around 0.04–0.05). The interpretation should consistently reflect the corrected analyses and distinguish exploratory findings from robust evidence.

**2. Multivariable modelling**

 The ordinal logistic regression models include a relatively large number of predictors considering the sample size (107 participants). This raises concerns regarding model overfitting, unstable coefficient estimates, and limited statistical power. The authors should better justify the modelling strategy, discuss the potential impact of overfitting, or consider a more parsimonious model.

**3. Causal interpretation**

 While the manuscript has improved considerably, some statements still imply causal relationships that cannot be inferred from a cross-sectional study. Expressions suggesting that one factor increases or causes another should be replaced by language describing associations. This is particularly important in the Discussion and Conclusions.

**4. Clinical interpretation**

 The discussion focuses primarily on statistical significance rather than the magnitude and clinical relevance of the observed associations. Greater emphasis on effect sizes and the potential clinical importance of the findings would strengthen the manuscript.

**Minor comments**

- The English language could benefit from additional editing to improve readability and consistency.
- Some discussion paragraphs remain lengthy and repetitive and could be condensed.
- Terminology should be standardized throughout the manuscript (e.g., haemodialysis/hemodialysis, CKD/ESRD).
- The Conclusions would benefit from a clearer distinction between the study findings and broader implications for clinical practice.

**Overall assessment**

 This study addresses an important clinical question and provides useful evidence from a setting where such data are scarce. The methodology is generally appropriate, and the manuscript has improved substantially following revision. Nevertheless, important concerns remain regarding the interpretation of the statistical analyses, the complexity of the multivariable models relative to the sample size, and the wording of some conclusions.

Is the work clearly and accurately presented and does it cite the current literature?

Partly

If applicable, is the statistical analysis and its interpretation appropriate?

Partly

Are all the source data underlying the results available to ensure full reproducibility?

Yes

Is the study design appropriate and is the work technically sound?

Partly

Are the conclusions drawn adequately supported by the results?

Partly

Are sufficient details of methods and analysis provided to allow replication by others?

Yes

Reviewer Expertise:

Primary Care, health systems

I confirm that I have read this submission and believe that I have an appropriate level of expertise to confirm that it is of an acceptable scientific standard, however I have significant reservations, as outlined above.

# Response

**Published:** 2026-07-26

**Authors:** Evelyn Goicochea Rios

**Affiliations:** Universidad Cesar Vallejo, Trujillo, La Libertad, Peru

**Major comments**

**1. Interpretation of statistical findings**

**Response: **We thank the reviewer for this important observation. Following this recommendation, we revised the statistical analysis by replacing the original multivariable models with more parsimonious ordinal logistic regression models (Tables 6 and 7). Consequently, the previous multiple-comparison issue that motivated the Bonferroni correction was no longer applicable, and the revised analyses provide a more robust and interpretable assessment of the independent associations. Accordingly, Tables 6 and 7 were replaced, and the Results, Discussion, Abstract, and Conclusions were revised to reflect the updated analyses. We also reduced the emphasis on isolated p-values and revised the interpretation of the findings to focus on the overall pattern, the magnitude of the associations, and the need for cautious interpretation given the cross-sectional design.

**2. Multivariable modelling**

 In response to this concern, we revised the multivariable analysis and replaced the original ordinal logistic regression models with more parsimonious models. The revised models included only the variables that met the predefined selection criteria, thereby reducing the number of predictors in relation to the sample size and improving model stability and interpretability.

 Accordingly, the revised models provide a more robust and clinically meaningful assessment of the factors independently associated with anxiety and depressive symptom severity.

**3. Causal interpretation**

**Response:** We carefully revised the manuscript to ensure that the language is fully consistent with the cross-sectional design of the study. Throughout the Discussion and Conclusions, statements implying causal relationships were replaced with language describing associations (e.g., *associated with*, *independently associated with*, *correlated with*, and *may be related to*), and causal expressions such as *increase*, *cause*, *lead to*, or *result in* were removed or rephrased where appropriate. We also revised the interpretation of the findings to emphasize that the observed relationships represent associations rather than causal effects. We believe these revisions provide a more accurate and methodologically appropriate interpretation of the results.

**4. Clinical interpretation**

**Response: **We revised the Discussion to place greater emphasis on the magnitude and potential clinical relevance of the observed associations, rather than focusing solely on statistical significance. Specifically, we incorporated interpretations of the effect sizes (e.g., weak or modest associations), discussed their potential clinical implications, and acknowledged that the observed demographic and biochemical factors are unlikely to fully explain anxiety and depressive symptoms, which are multifactorial in nature. We also revised the Abstract and Conclusions to ensure that the interpretation of the findings reflects both their statistical and clinical significance.

 These revisions were intended to provide a more clinically meaningful interpretation of the results while acknowledging the limitations imposed by the cross-sectional design and the relatively small sample size.

**Minor comments**

- The English language could benefit from additional editing to improve readability and consistency.
- **Response:** The manuscript was thoroughly revised to improve grammar, readability, sentence structure, and consistency throughout the text.
- Some discussion paragraphs remain lengthy and repetitive and could be condensed.
- **Response:** The Discussion was carefully edited to eliminate redundancy, condense lengthy paragraphs, and improve the logical flow while preserving the scientific content.
- Terminology should be standardized throughout the manuscript (e.g., haemodialysis/hemodialysis, CKD/ESRD).
- **Response:** Terminology was standardized throughout the manuscript to ensure consistency. In particular, uniform terminology was adopted for dialysis modalities and kidney disease classification.
- The Conclusions would benefit from a clearer distinction between the study findings and broader implications for clinical practice.
- **Response:** The Conclusions were revised to clearly separate the principal findings of the study from their clinical implications, while avoiding causal inferences and maintaining consistency with the cross-sectional design.

# Reviewer report: Reviewer response for version 1

**DOI:** 10.5256/f1000research.197321.r480865

**Published:** 2026-05-28

**Authors:** Hanoch Livneh

**Affiliations:** 1 Portland State University, Portland, Oregon, USA

The authors of “Prevalence and factors associated with depression and anxiety in patients with CKD” describe a cross-sectional, correlational study, attempting to explore both the prevalence of anxiety and depression among CKD patients, as well the association between these two self-reported psychological conditions and a wide range of biochemical markers.

 The study is generally well conceived, its data are (mostly) accurately analyzed, and its findings are unambiguously reported.

 There are, however, several areas that need to be further addressed. These include:

1. Introduction (p.3):

- 2 ^nd^ paragraph (Pa): depression is not necessarily a product (effect) of eating disorders (and mental illness) but may also be the cause of such conditions.
- 6 ^th^ Pa: The authors “flood” this paragraph with relentless bits of data, making for difficult reading, and at times, unclear findings.
- 8 ^th^ Pa:  The HADS-related paragraph should be placed in the Methods section.

1. Patients and methods (pp.4-5):

- 1 ^st^ Pa: This is not a prospective cohort research design. Data were obtained at a single point in time.
- 3rd Pa: The authors should report psychometric characteristics of the HADS in both the formal/original scale manual as well as in their own study.
- 4 ^th^ Pa (and later Pa’s): change “the instrument was applied” to “the measure was administered”.
- 8 ^th^ Pa: The Pa ends with the digit “1”. It is unclear as to its meaning.

1. Results (pp.5-9):

- 5 ^th^ and 6 ^th^ Pa’s: The authors should stay consistent. Either use “participants showed” across the various measured traits or “participants did not show”. Mixing the two often results in unclear statements.
- Pp.8-9: (Results reported for Table 7; and in other segments of the manuscript): First, the authors often make the mistake of attributing causal relationships to their cross-sectional findings. For example, “the presence of sleep disorders significantly increased the probability of higher levels of depression”. It can just as well be argued that depression leads to sleep disorders. Second, another conclusionary concern is the authors’ assigning disproportional value to “barely significant” alpha levels (e.g., in Table 7, p=.046). The authors should be reminded that they used a large number of predictors (up to n=17) in their models and conducted numerous comparisons and statistical tests per both their correlational matrices, as well as in their ordinal regression models. They should have adjusted their alpha levels accordingly, to minimize risk of inflated Type I (false positive) error levels (e.g., use of Bonferroni correction procedure; dividing the standard p=.05 by the numbers of attempted comparisons).

1. Discussion (pp.9-16):

- P. 14, 1 ^st^ Pa: Again, it is important for the authors to recognize that it would be inaccurate to assume “causal” relationships derived from their findings, in particular those assumed between unemployment and depression. In fact, a third “hidden variable” interpretation maybe be just as plausible (e.g., diabetes or other medical conditions, or treatment-related variable influencing both depression and functioning (unemployment). The same rationale can be applied to the discussion in Pa 4, where the authors seek to introduce a causal link between sleep disorders and anxiety.
- P. 14 (bottom Pa): Again, the authors should be cautioned about concluding a significant statistical relationship between hemoglobin and anxiety (p=.042).
- P. 16 (Limitations): This 4-line subsection needs to be rewritten and expanded to address several limitations inherent in the study. Additionally, it is important to clarify that the study is not prospective, but rather cross-sectional. Adding a sentence about the need for a longitudinal research design, to confirm many of the findings, should also be included. Finally, the limitations imposed (e.g., the findings’ statistical reliability) because of the small sample size and the low ratio of variables-to-participants should also be acknowledged.
- P. 16: Either in the “limitations” subsection or in a separate subsection (Recommendations for future research), the authors should consider the fact that the findings from their study could be fine-tuned by adopting a mediation model where variables such as “coping strategies” and /or “personality traits” mediate the relationships between medical/biochemical predictors and psychosocial outcomes (in their case, anxiety and depression).
- P. 16 (Conclusions): As previously noted, the authors need to “tone down” their causal claims and acknowledge the limitation of their findings. In addition, I would recommend strengthening the conclusion section by addressing some related pertinent findings in the context of the larger picture of the link between psychological reactions and CKD. As it reads, at present, this section is merely a condensed rehash of the Discussion section, with only a limited view of the bigger picture and of any insightful points the reader may take away from this study. 

Is the work clearly and accurately presented and does it cite the current literature?

Partly

If applicable, is the statistical analysis and its interpretation appropriate?

Partly

Are all the source data underlying the results available to ensure full reproducibility?

Yes

Is the study design appropriate and is the work technically sound?

Yes

Are the conclusions drawn adequately supported by the results?

Partly

Are sufficient details of methods and analysis provided to allow replication by others?

Yes

Reviewer Expertise:

psychosocial adaptation to chronic illness and disability

I confirm that I have read this submission and believe that I have an appropriate level of expertise to confirm that it is of an acceptable scientific standard, however I have significant reservations, as outlined above.

# Response

**Published:** 2026-06-23

**Authors:** Evelyn Goicochea Rios

**Affiliations:** Universidad Cesar Vallejo, Trujillo, La Libertad, Peru

Dear reviewer:

 We are very grateful for your comments, which have helped us to improve the article. Our response is set out below

1. Introduction (p.3):

- 2 ^nd^ paragraph (Pa): depression is not necessarily a product (effect) of eating disorders (and mental illness) but may also be the cause of such conditions.
- ***Response: **We agree with the comment. We have quoted the WHO’s definition of depression (reference 4), so we have changed ‘is characterised by’ to ‘is defined as’.*
- 6 ^th^ Pa: The authors “flood” this paragraph with relentless bits of data, making for difficult reading, and at times, unclear findings.
- ***Response:** The wording of this paragraph was amended:*
* The association between depression and various sociodemographic variables in patients with CKD has been documented, ^10-13 ^including gender, educational attainment, age group, employment status and duration of CKD ^10^ and monthly income; ^13^ other authors consider  that  family support, ^10,11^ social support and marital status ^12^ are associated with clinical depression, as are a negative perception of the disease and low self-esteem. ^10^ It is also noted that socio-economic variables affect the quality of life of people with CKD. ^14^*
- 8 ^th^ Pa:  The HADS-related paragraph should be placed in the Methods section.
- ***Response: **The paragraph has been moved to ‘Methods’ .-(see instrument)*
- Patients and methods (pp.4-5):
- 1 ^st^ Pa: This is not a prospective cohort research design. Data were obtained at a single point in time.
- ***Response: ** It was changed in line with the suggestion: cross-sectional-correlational*
- 3rd Pa: The authors should report psychometric characteristics of the HADS in both the formal/original scale manual as well as in their own study.
- ***Response: ** The information on the original HADS and from a Peruvian study was included. For our study, we used the HADS as a widely validated instrument.*
- 4 ^th^ Pa (and later Pa’s): change “the instrument was applied” to “the measure was administered”.
- ***Response: **It was changed in line with the suggestion*
- 8 ^th^ Pa: The Pa ends with the digit “1”. It is unclear as to its meaning.
- ***Response: ** The number 1 at the end of the paragraph was removed.*
- Results (pp.5-9):
- 5 ^th^ and 6 ^th^ Pa’s: The authors should stay consistent. Either use “participants showed” across the various measured traits or “participants did not show”. Mixing the two often results in unclear statements.
- ***Response: ** It was changed in line with the suggestion.*
- Pp.8-9: (Results reported for Table 7; and in other segments of the manuscript): First, the authors often make the mistake of attributing causal relationships to their cross-sectional findings. For example, “the presence of sleep disorders significantly increased the probability of higher levels of depression”. It can just as well be argued that depression leads to sleep disorders. Second, another conclusionary concern is the authors’ assigning disproportional value to “barely significant” alpha levels (e.g., in Table 7, p=.046). The authors should be reminded that they used a large number of predictors (up to n=17) in their models and conducted numerous comparisons and statistical tests per both their correlational matrices, as well as in their ordinal regression models. They should have adjusted their alpha levels accordingly, to minimize risk of inflated Type I (false positive) error levels (e.g., use of Bonferroni correction procedure; dividing the standard p=.05 by the numbers of attempted comparisons).
- ***Response: **The Bonferroni correction procedure was applied.*

1. Discussion (pp.9-16):

- P. 14, 1 ^st^ Pa: Again, it is important for the authors to recognize that it would be inaccurate to assume “causal” relationships derived from their findings, in particular those assumed between unemployment and depression. In fact, a third “hidden variable” interpretation maybe be just as plausible (e.g., diabetes or other medical conditions, or treatment-related variable influencing both depression and functioning (unemployment). The same rationale can be applied to the discussion in Pa 4, where the authors seek to introduce a causal link between sleep disorders and anxiety.
- ***Response: **It was changed in line with the suggestion.*
* The Bonferroni correction was applied.*
- P. 14 (bottom Pa): Again, the authors should be cautioned about concluding a significant statistical relationship between hemoglobin and anxiety (p=.042).
- ***Response: **We have removed the term ‘significant’ from the interpretation of these and other results.*
- P. 16 (Limitations): This 4-line subsection needs to be rewritten and expanded to address several limitations inherent in the study. Additionally, it is important to clarify that the study is not prospective, but rather cross-sectional. Adding a sentence about the need for a longitudinal research design, to confirm many of the findings, should also be included. Finally, the limitations imposed (e.g., the findings’ statistical reliability) because of the small sample size and the low ratio of variables-to-participants should also be acknowledged.
- P. 16: Either in the “limitations” subsection or in a separate subsection (Recommendations for future research), the authors should consider the fact that the findings from their study could be fine-tuned by adopting a mediation model where variables such as “coping strategies” and /or “personality traits” mediate the relationships between medical/biochemical predictors and psychosocial outcomes (in their case, anxiety and depression).
- ***Response: **This section has been rewritten. Please see the changes in the new version.*
- P. 16 (Conclusions): As previously noted, the authors need to “tone down” their causal claims and acknowledge the limitation of their findings. In addition, I would recommend strengthening the conclusion section by addressing some related pertinent findings in the context of the larger picture of the link between psychological reactions and CKD. As it reads, at present, this section is merely a condensed rehash of the Discussion section, with only a limited view of the bigger picture and of any insightful points the reader may take away from this study. 
- ***Response:**This section has been rewritten.Please see the changes in the new version.*
